# Chromosome-level genome of the globe skimmer dragonfly (*Pantala flavescens*)

**DOI:** 10.1093/gigascience/giac009

**Published:** 2022-04-04

**Authors:** Hangwei Liu, Fan Jiang, Sen Wang, Hengchao Wang, Anqi Wang, Hanbo Zhao, Dong Xu, Boyuan Yang, Wei Fan

**Affiliations:** Guangdong Laboratory for Lingnan Modern Agriculture (Shenzhen Branch), Genome Analysis Laboratory of the Ministry of Agriculture and Rural Affairs, Agricultural Genomics Institute at Shenzhen, Chinese Academy of Agricultural Sciences, Shenzhen, Guangdong 518120, China; Guangdong Laboratory for Lingnan Modern Agriculture (Shenzhen Branch), Genome Analysis Laboratory of the Ministry of Agriculture and Rural Affairs, Agricultural Genomics Institute at Shenzhen, Chinese Academy of Agricultural Sciences, Shenzhen, Guangdong 518120, China; Guangdong Laboratory for Lingnan Modern Agriculture (Shenzhen Branch), Genome Analysis Laboratory of the Ministry of Agriculture and Rural Affairs, Agricultural Genomics Institute at Shenzhen, Chinese Academy of Agricultural Sciences, Shenzhen, Guangdong 518120, China; Guangdong Laboratory for Lingnan Modern Agriculture (Shenzhen Branch), Genome Analysis Laboratory of the Ministry of Agriculture and Rural Affairs, Agricultural Genomics Institute at Shenzhen, Chinese Academy of Agricultural Sciences, Shenzhen, Guangdong 518120, China; Guangdong Laboratory for Lingnan Modern Agriculture (Shenzhen Branch), Genome Analysis Laboratory of the Ministry of Agriculture and Rural Affairs, Agricultural Genomics Institute at Shenzhen, Chinese Academy of Agricultural Sciences, Shenzhen, Guangdong 518120, China; Guangdong Laboratory for Lingnan Modern Agriculture (Shenzhen Branch), Genome Analysis Laboratory of the Ministry of Agriculture and Rural Affairs, Agricultural Genomics Institute at Shenzhen, Chinese Academy of Agricultural Sciences, Shenzhen, Guangdong 518120, China; Guangdong Laboratory for Lingnan Modern Agriculture (Shenzhen Branch), Genome Analysis Laboratory of the Ministry of Agriculture and Rural Affairs, Agricultural Genomics Institute at Shenzhen, Chinese Academy of Agricultural Sciences, Shenzhen, Guangdong 518120, China; Guangdong Laboratory for Lingnan Modern Agriculture (Shenzhen Branch), Genome Analysis Laboratory of the Ministry of Agriculture and Rural Affairs, Agricultural Genomics Institute at Shenzhen, Chinese Academy of Agricultural Sciences, Shenzhen, Guangdong 518120, China; Guangdong Laboratory for Lingnan Modern Agriculture (Shenzhen Branch), Genome Analysis Laboratory of the Ministry of Agriculture and Rural Affairs, Agricultural Genomics Institute at Shenzhen, Chinese Academy of Agricultural Sciences, Shenzhen, Guangdong 518120, China

## Abstract

**Background:**

The globe skimmer dragonfly (*Pantala flavescens*) is a notable Odonata insect distributed in nature fields and farmlands worldwide, and it is commonly recognized as a natural enemy because it preys on agricultural pests and health pests. As one of the sister groups of winged insects, odonatan species are key to understanding the evolution of insect wings.

**Findings:**

We present a high-quality reference genome of *P. flavescens*, which is the first chromosome-level genome in the Palaeoptera (Odonata and Ephemeroptera). The assembled genome size was 662 Mb, with a contig N50 of 16.2 Mb. Via Hi-C scaffolding, 648 Mb (97.9%) of contig sequences were clustered, ordered, and assembled into 12 large scaffolds, each corresponding to a natural chromosome. The X chromosome was identified by sequence coverage depth. The repetitive sequences and gene density of the X chromosome are similar to those of autosomal sequences, but the X chromosome shows a much lower degree of heterozygosity. Our analysis shows that the effective population size experienced 3 declining events, which may have been caused by climate change and environmental pollution.

**Conclusions:**

The genome of *P. flavescens* provides more information on the biology and evolution of insects and will help for the use of this species in pest control.

## Background

The use of predatory insects has resulted in enormous economic and ecological benefits [1]. There have been many successful cases, such as Vedalia ladybird beetle *Novius cardinalis* (Mulsant, 1850) in control of cottony cushion scale [2], and *Trichogramma* spp. used for control of Lepidoptera pests [3]. Many odonatan species are considered important natural enemies of many insect pests such as *Anopheles* mosquitoes, flies, and gnats [4]. The globe skimmer dragonfly *Pantala flavescens* (NCBI:txid185825), a member of the Libellulidae (Insecta: Odonata), occurs worldwide and contributes to control of agricultural pests and health pests [5]. Previous studies have revealed that *P. flavescens* is the most widespread species of the Odonata, widely distributed throughout the tropics and many temperate areas. *P. flavescens* has a powerful capability to migrate several thousand kilometers [6–8], and transoceanic migration of *P. flavescens* >10,000 km often occurs every October–December. However, *P. flavescens* has exhibited drastic population decreases in the past several hundred years owing to environmental pollution and human activities [5].

Odonata are diverse, numerous, commonly observed, and species rich, and >6,000 species have been described [9, 10]. These insects have strikingly colourful bodies, giant compound eyes, and an active flying ability. Odonata consists of 2 main suborders, Anisoptera (dragonflies) and Zygoptera (damselflies), which show significant discrepancies. Dragonflies are generally robust, and their wings spread flat at rest, while damselflies are slender and hold their wings over their abdomen at rest [9]. Odonatan species date to the Carboniferous (360–290 million years ago [Mya]) according to many complete and well-preserved fossil records [11]. Odonata and Ephemeroptera (mayflies) are members of Palaeopteran insects, which are the first winged insect and the sister group of Neopterans [12]. The evolution of wings in insects is a major event because the appearance of wings has promoted insects to become the largest and most abundant animal taxon on Earth [13, 14].

Genomic resources for Odonata available in public databases are much fewer than for other orders of insects such as dipteran, lepidopteran, hymenopteran, and blattaria. Only 4 genomes of odonatan species (*Rhinocypha anisoptera, Calopteryx splendens, Ladona fulva, Ischnura elegans*) with low continuity have been released [15–17], and a high-quality genome of odonatan species is necessary for insect research. Recent advances in circular consensus sequencing (CCS), which can generate highly accurate (99.8%), long, high-fidelity (HiFi) reads [18], combined with sophisticated assembly software such as Hifiasm (Hifiasm, RRID:SCR_021069) [19] and HiCanu [20], provide a promising way to generate high-quality reference genome sequence. In this study, we sequenced the genome of *P. flavescens*, as a representative odonatan species, with HiFi technology, and obtained a chromosome-level genome assembly, along with an integral comprehensive gene set. The high-quality genomic data enable the identification and analysis of the sex chromosome, and the inferring of evolution and demographic history.

## Materials and Methods

### Insect arrest and genomic sequencing

Male and female *P. flavescens* adults were collected at ShenZhen Station of the Chinese Academy of Agricultural Sciences, Guangdong Province, China. Insects were removed from the intestine to avoid contamination from bacterial and prey genomes, cleaned using 30% ethanol and ddH_2_O, and then immersed in liquid nitrogen.

For Illumina sequencing, a short paired-end DNA library with a 400-bp insert size from a female adult and a male adult *P. flavescens* was constructed using standard Illumina protocols and sequenced on an Illumina HiSeq 2500 platform (Illumina HiSeq 2500 System, RRID:SCR_016383). For Pacific Biosciences (PacBio) HiFi sequencing, 2 libraries with ∼15 kb insert sizes were constructed from a female adult using PacBio SMRT. PacBio long reads were sequenced using 2 cells on a PacBio Sequel II system (PacBio Sequel II System, RRID:SCR_017990). A total of 831 Gb of subreads were generated with an N50 of 14.3 kb. Consensus reads (CCS reads) were generated using ccs software v.3.0.0 [21] with the following parameters: –min-passes 0 –min-rq 0.99 –min-length 100 –max-length 50,000. The total CCS read yield was 50 Gb, with a read length of 14.5 kb.

Total RNA was extracted from a female adult and a male adult and then mixed to generate the libraries. Synthesized full-length complementary DNAs were then used to prepare 3 20-kb SMRTbell template libraries for sequencing on a PacBio Sequel instrument.

### Genome assembly and quality assessment

The PacBio reads were assembled using Hifiasm (0.12-r304) with the following parameters: -l 1 -s 0.7. This resulted in 196 contigs with a total length of ∼691 Mb and a contig N50 of 15.8 Mb. To filter duplicate contigs in the assembly, purge_dups (v1.2.3) (purge dups, RRID:SCR_021173) [21] was used with the following parameters: −2 -a 50. This resulted in a purged primary assembly with a total length 662 Mb and a contig N50 of 16.2 Mb.

The quality of the assembly was evaluated using BUSCO v5.2.2 (BUSCO, RRID:SCR_015008) [22] based on OrthoDB v10 of Insecta (OrthoDB, RRID:SCR_011980). Iso-seq full-length transcripts were also used to evaluate the accuracy of the genome. First, raw Iso-seq data were subjected to read quality filtering, read clustering, consensus calling, and polishing using SMRT Analysis v.2.3 (SMRT-Analysis, RRID:SCR_002942) [22] and then assembled into high-quality and full-length transcripts. These full-length transcripts were then aligned to the genome using GMAP (version 2020–10-27) (GMAP, RRID:SCR_008992) [23] to evaluate the structural accuracy of the assembly.

### Genome scaffolding

A total of 170 Gb of Hi-C paired-end reads were generated from a female adult, with a Q30 of 92.28%. After quality control, the clean reads were mapped to the genome by Bowtie2 (v2.3.4.3) (Bowtie 2, RRID:SCR_016368), and then HiC-Pro (v2.11.0) (HiC-Pro, RRID:SCR_017643) was used to generate an alignment file to detect valid alignments and filter multiple hits and singletons. Finally, LACHESIS (LACHESIS, RRID:SCR_017644) [24] was used to cluster, order, and orient the contigs.

### Detection of X chromosome

Clean Illumina female and male read data were mapped to the chromosome-level genome with BWA, and the sequencing depth was calculated with SAMtools (SAMTOOLS, RRID:SCR_002105). The autosomes should have equal coverage, while the X chromosome should show approximately half coverage in males.

### Genome annotation

A *de novo* repeat library was constructed with RepeatModeler (v1.0.8) (RepeatModeler, RRID:SCR_015027) (parameters: -engine ncbi-database). RepeatMasker (RepeatMasker, RRID:SCR_012954) was then used to identify transposable element (TE) repeats by combining the contents of the *de novo* repeat library and a TE database (Dfam 3.0, RRID:SCR_021168 and RepBase 20170127, RRID:SCR_021169). To avoid protein-coding genes being marked as repeats, we aligned the 260 repeat sequences of “unknown” type to NR database by blastx (v2.7.1+) (BLASTX, RRID:SCR_001653) using 1e^−5^ as cut-off, and 53 of them were found to have homology with known non-TE protein-coding genes, which were filtered out of the RepeatModeler *de novo* library. Repeatmasker was then used to find TEs based on the filtered *de novo* TE library.

*De novo* prediction of coding genes was performed using repeat-masked genome sequences. The gene model parameters of AUGUSTUS (Augustus, RRID:SCR_008417) [25] were trained using Iso-seq full-length transcripts. For homology-based prediction, the protein sequences of odonatan species were downloaded from the NCBI and UniProt (UniProt, RRID:SCR_002380) databases and mapped to the genome with exonerate (version 2.4.0) (Exonerate, RRID:SCR_016088), and incomplete gene models were filtered and removed. Quality-controlled reads from 2 RNA libraries (accession Nos. SRR1184263 and SRR1184243) were mapped to the genome using Bowtie2, and StringTie (StringTie, RRID:SCR_016323) was used to construct gene prediction models. Iso-seq full-length transcripts were mapped to the genome with GAMP. Finally, all the genes predicted with the 4 approaches were integrated with EVidenceModeler (EVidenceModeler, RRID:SCR_014659) [26] to generate high-confidence gene sets (Supplementary Table S2).

To evaluate the accuracy of the gene sets, the coverage of highly conserved genes was assessed using BUSCO based on OrthoDB v10 of Insecta. For gene functional annotation, we aligned the protein sequences of genes with the KEGG (KEGG, RRID:SCR_012773), eggNOG (eggNOG, RRID:SCR_002456), NR, and UniProt (SwissProt) databases in Diamond, with 1e^–5^ used as a cut-off, and obtained the best hit. We also used InterProScan (v5.38–76.0) (InterProScan, RRID:SCR_005829) to search the InterPro (InterPro, RRID:SCR_006695) database to identify motifs and domains.

### Evolutionary analysis

Fourteen sequenced arthropoda species, including *Parasteatoda tepidariorum* [27], *Strigamia maritima* [28], *Daphnia pulex* [29], *Folsomia candida* [30], *Catajapyx aquilonaris, L. fulva* [16], *P. flavescens, Cloeon dipterum* [31], *Zootermopsis nevadensis* [32], *Acyrthosiphon pisum* [33], *Drosophila melanogaster* [34], *Danaus plexippus* [35], *Tribolium castaneum* [36], and *Apis mellifera* [37], were used to infer orthologous genes in OrthoFinder (OrthoFinder, RRID:SCR_017118) [38] with the default parameters. The protein sequences of single-copy genes from each species were aligned in MUSCLE (v3.8.1551) (MUSCLE, RRID:SCR_011812) [39] and then concatenated into 1 supersequence. RAxML (version 8.2.12) (RAxML, RRID:SCR_006086) [40] was subsequently used to construct a phylogenetic tree based on the concatenated supersequence with the PROTGAMMALGX model. Divergence times among species were calculated in MCMCtree (PAML package, v. 4.9) (PAML, RRID:SCR_014932) [41]. The calibration times were set according to the data in a previous article: a minimum of 308 Mya and maximum of 366 Mya for *D. melanogaster* and *A. pisum*, a minimum of 413 Mya and maximum of 483 Mya for *D. melanogaster* and *C. aquilonaris*, a minimum of 413 Mya and maximum of 483 Mya for *D. melanogaster* and *C. aquilonaris*, and a minimum of 452 Mya and maximum of 557 Mya for *D. pulex* and *A. pisum* [12]. The phylogenetic tree and gene results were displayed and annotated using Evolview [42].

### Demographic history

Raw reads were processed to obtain clean reads using fastp (0.20.0) (fastp, RRID:SCR_016962) [43]. The quality-controlled reads were mapped to the genome using BWA (version 0.7.15) (BWA, RRID:SCR_010910), with the default parameters. SAMtools (version 1.4) was used for sorting, and Picard (v.2.17.0) (Picard, RRID:SCR_006525) was used to remove duplicates. Single-nucleotide polymorphism (SNP) calling was then performed using the GATK (4.0.4.0) (GATK, RRID:SCR_001876) HaplotypeCaller. To obtain high-quality SNPs, we initially used the GATK hard filter to remove the merged VCF data with the following options: QD ≥ 2.0 && FS ≤ 60.0 && MQ ≥ 40.0 && MQRankSum ≥ −12.5 && ReadPosRankSum ≥ −8.0. SNPs present on the X chromosomes were excluded to avoid potential bias by sex. Female and male data were used to estimate demographic history using SMC++ [44]. We used a mutation rate of 1 × 10^−9^ per generation per year, and 1 generation per year.

## Results

### Chromosome-level genome assembly of *P. flavescens*

To obtain a high-quality genome, 50 Gb (80-fold) of HiFi reads (Supplementary Table S1) from an adult female were generated with a read N50 length of 14.5 kb. Before genome *de novo* assembly, a genome survey based on *k*-mer frequency showed that the genome size is 663 Mb (Supplementary Fig. S1). The total length of the genome assembly produced by Hifiasm is ∼691 Mb, comprising 196 contigs with an N50 size of 15.8 Mb. This genome assembly is slightly larger than the estimated genome size, which may result from genome heterozygosity. Using purge_dups to reassign allelic contigs, a reference assembly was generated comprising 99 contigs with a total length of 662 Mb (Table 1), which is comparable to the estimated genome size. The contig N50 size of the genome assembly is 16.2 Mb, and the longest contig is 41.7 Mb [45]. The completeness of the draft genome was evaluated via BUSCO [22]. Of the 1,367 single-copy orthologous genes in the BUSCO insecta_odb10 database, 1,325 (96.9%) were identified in this draft genome, including 1,280 (93.6%) complete and single-copy BUSCO genes and 45 (3.3%) complete and duplicated BUSCO genes. A total of 45,601 transcripts produced using PacBio single-molecule long-read sequencing were mapped to the genome assembly with GAMP (version 2020–10-27) [23], and 99.5% (45,366) were mapped successfully with an average identity of 99.1% and an average coverage of 98.4%. These results also reflect the high accuracy of our assembly.

**Table 1:** : Major indicators of the *P. flavescens* genome

Indicator type	Assembly feature	Value
	Estimated genome size	663 Mb
Contigs	Total size of contigs	662 Mb
	Counts of contigs	99
	N50 size	16.2 Mb
Scaffolds	Total size of scaffolds	648 Mb
	Counts of scaffolds	12
	N50 size	53 Mb
Genome annotation	Total gene number	15,354
	Mean CDS length	1,528
	Mean exon number	7.1
Repeat annotation	SINEs	35 kb
	LINEs	13 Mb
	LTR	1.2 Mb
	DNA	31 Mb
	Unclassified	73 Mb

LINE: long interspersed nuclear element; LTR: long terminal repeat; SINE: short interspersed nuclear element.

**Figure 1:**
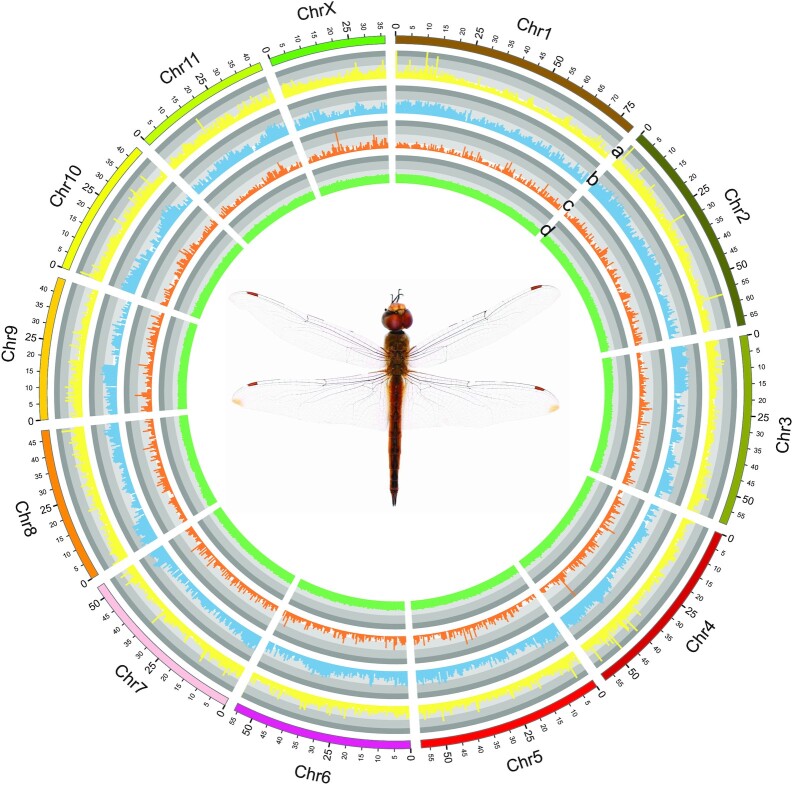
: The genome landscape of *Pantala flavescens*. Circular representation of the chromosomes. Tracks a–d represent the distribution of tandem repeat density, transposable element (TE) density, gene density, and GC density, respectively, with densities calculated in 500-kb windows.

The LACHESIS pipeline was used to anchor and orient 648 Mb (97.6%) of contigs to 12 pseudochromosomes (Table 1, Fig. 1, Supplementary Fig. S2), which corresponded to the 12 chromosomes. The N50 size of this chromosome-level genome was 53 Mb, with the longest 79 Mb and the shortest 36 Mb. Approximately 80% of the 31 unanchored contigs constituted repetitive sequences, indicating that most unanchored contigs were repeat fragments.

A total of 117 Mb (17.8% of the nuclear genome) of interspersed repeats were identified in the *P. flavescens* genome (Table 1). Among them, DNA (31 Mb), long interspersed nuclear elements (LINEs) (13 Mb), and LTRs (1.2 M) were the major types of TEs. A total of 15,354 protein-coding gene models were predicted by EVidenceModeler (Supplementary Table S2), with a mean CDS length of 1,528 bp and a mean exon number of 7.1, comparable to that of other published insects. In terms of evaluating the completeness of the predicted gene sequences with the sequences of 1,367 BUSCO genes from insecta_odb10, 1,352 BUSCOs (98.9%) were determined to be complete, which is better than that of the genome (96.9%). Compared to those of the other 2 Palaeopteran species, *C. dipterum* and *L. fulva*, the BUSCO complete ratio of *P. flavescens* is the highest (Table 2). For functional annotation, 12,995 (85%), 12,417 (81%), and 10,346 (67%) genes have homologous sequences in the NR, Uniprot, and KEGG databases, respectively. In addition, 13,240 (86%) genes were annotated by InterProScan. In summary, 14,024 (91%) genes were annotated by ≥1 functional databases or methods (Table S3).

**Table 2:** : BUSCO assessment of gene sets of *P. flavescens* and other Insecta species

Species	C (%)	S (%)	D (%)	F (%)	M (%)
*A. mellifera*	99.1	61.4	37.7	0.2	0.7
*T. castaneum*	99.0	98.6	0.4	0.3	0.7
*D. plexippus*	99.5	97.7	1.8	0.2	0.3
*D. melanogaster*	99.2	98.3	0.9	0.6	0.2
*A. pisum*	95.7	89.6	6.1	1.0	3.3
*F. occidentalis*	98.8	97.1	1.7	0.7	0.5
*Z. nevadensis*	98.0	97.4	0.6	0.6	0.8
*C. dipterum*	95.2	91.8	3.4	1.0	3.8
*P. flavescens*	98.9	95.5	3.4	0.0	1.1
*L. fulva*	81.7	79.7	2.0	13.0	5.3
*C. aquilonaris*	85.0	83.6	1.4	6.7	8.3

C: Complete BUSCOs; S: Complete and single-copy BUSCOs; D: Complete and duplicated BUSCOs; F: Fragmented BUSCOs; M Missing BUSCOs.

### X chromosome identification

**Figure 2:**
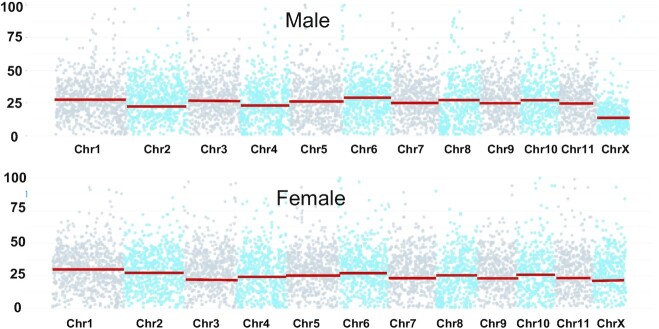
: X chromosome identification. Male and female sequence depth plotted in 500 bp of every chromosome. Red line represents the mean sequencing depth.

Sex chromosomes evolved from autosomes and play important roles in tissue development, mating, and speciation [46–48]. A previous study showed that *P. flavescens* has an X0 sex determination, in which females possess 2 X chromosomes and males possess 1 X chromosome [49]. The X chromosome was determined by mapping resequencing data from males and females to the genome assembly. In males, the mean depth of chr12 was almost half that of the other chromosomes, and the mean depths of all chromosomes in females were similar (Fig. 2). Therefore, chr12, which has a total length of 36.2 Mb and contains 6 contigs, was designated as the X chromosome. This is the shortest chromosome and is consistent with karyotype [49]. X0 sex determination has also been discovered in Orthoptera and some Hemiptera species such as aphids and psyllids [50, 51]. In aphids, the characteristics of the X chromosome are different from those of the autosomal chromosomes. The X chromosome of *A. pisum* (aphids) is enriched in repetitive sequences, and the gene density is lower than that of the autosomes [52]. However, in *P. flavescens*, repeat sequences constitute 20.8% of the X chromosome, comparable to that of the autosomal sequences (17.6%), and the gene density is also comparable between the X chromosome and autosomals.

The heterozygosity of the *P. flavescens* genome was estimated by heterozygous SNPs, and a sharp decrease in heterozygosity was noticed. The heterozygosity of the X chromosome is 0.5%, which is less than half that of the autosomes ( 1.3%). The evolution of sex chromosomes is poorly understood in Palaeopteran insects. Here, we present the first X chromosome sequence information in Palaeopteran insects, which may promote research on the evolution of sex chromosomes.

### The population size decline

To investigate the genome evolutionary history of *P. flavescens*, gene family members were subjected to clustering analysis using *P. flavescens* and 14 other arthropoda species, including chelicerates, myriapods, crustaceans, and hexapods [16]. From the gene family clustering results, 447 single-copy orthologs shared between *P. flavescens* and 14 other arthropoda species were used for phylogenetic construction and species divergence time estimation, representing arthropod evolution spanning >500 million years. We estimated that *P. flavescens* and *L. fulva* shared a common ancestor at ∼125 Ma, and divergence of *P. flavescens* and *C. dipterum* was estimated to have occurred at ∼420 Ma (Fig. 2a). Our phylogenetic tree and estimated divergence time are mostly consistent with previous arthropod phylogenetic studies [12, 16].

**Figure 3:**
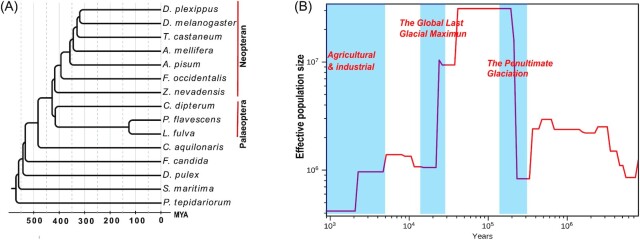
: Genome evolution of *P. flavescens*. (A) Phylogenetic relationships and gene orthology of *P. flavescens* with other arthropoda species. The maximum likelihood phylogenomic tree was calculated on the basis of 447 single-copy universal genes. (B) Demographic history of *P. flavescens* reconstructed from 2 adult resequencing data. Blue frame represents the geological events.

Effective population size (*N*_e_) is considered a pivotal parameter in population genetics and has been applied in the analysis of evolutionary biology, conservation genetics, and animal molecular breeding because it measures genetic drift and inbreeding in real-world populations. A decline in population size comes with a loss of genetic diversity and an increase in inbreeding [53, 54], which is harmful for adaptation to complex environments. Global climate change has been recognized to profoundly reshape animal population demographics [17, 55]. Monitoring the changes in effective population size over time for wild species is important for understanding genetic health and evaluating the risk of extinction. Here, we estimated the history of population sizes using SMC++ [44], and identified 3 events in which the population declined severely (Fig. 3B). The most ancient decline occurred during the Penultimate Glaciation (0.30–0.13 Mya), and afterwards, population expansion occurred. The second declination occurred at the Last Glacial Maximum (∼26.5–19 kya) [56], which is the most recent period of extreme cold. Many wild species, such as pandas, buffaloes, and ibis, experienced significant population declines during these 2 periods [57–59]. The results also revealed population declines in the past several thousand years (Fig. 3B), which might be due to recent human exploitation and habitat loss. Evidence has indicated that, while global climate change has been the primary driver of population fluctuations for millions of years, human activities likely underlie recent population divergence and severe decline. Further genome resequencing of *P. flavescens* will provide more detailed insights into the demographic history.

## Discussion

Here, we present a 662-Mb chromosome-scale reference genome of *P. flavescens* obtained using PacBio HiFi and Hi-C data, which is the first chromosome-scale reference genome in the Palaeoptera, and we also identified sex chromosomes in the Palaeopterans for the first time. The high BUSCO complete ratio and RNA mapping percentage confirmed the high quality of the reference genome assembly. Our analysis showed 3 events in which the population declined severely. The key features of odonatan species, including their ancient phylogenetic position, strong migration capability, and complex living environment, make *P. flavescens* a potential model of insect species. The genome and gene data of *P. flavescens* would facilitate the exploration of many important evolutionary, developmental, and physiological studies on insects. Furthermore, *P. flavescens* preys on many agricultural and sanitary pests; this species has great potential for use in pest control. Our data and results will also help the development of pest management technologies.

## Data Availability

All the raw sequencing data and genome data in this study are available in NCBI as a BioProject under accession PRJNA763384. Genomic sequence reads have been deposited in the SRA database with accessions SRR15902700, SRR15902700, SRR15910096, SRR15910131. Transcriptome sequence reads have been deposited in the SRA database with accession SRR15914636. Raw data of Hi-C have been deposited in the SRA database with accession SRR15910100. Genome assembly is available in DNA Data Bank of Japan/ENA/GenBank under the accession JAIUJI010000000. Supporting data and materials are available in the* GigaDB* database [45]. All the raw sequencing data and genome data in this study are available to community.

## Additional Files

**Supplementary Table S1:** Sequencing statistics

**Supplementary Table S2:** Statistics of gene predictions from different gene prediction methods

**Supplementary Table S3:** Functional annotation of gene set

**Supplementary Figure S1:** Estimated genome size using 17-mer. We used error-corrected Illumina reads from the short insert-size libraries to calculate the *k*-mer frequency. The peak depth of this curve was 80. The estimated genome size of *Pantala flavescens* was 663 Mb.

**Supplementary Figure S2:** HiC heat map of LACHESIS. A genome-wide contact matrix from Hi-C data between each pair of the 12 chromosomes using a 1-Mb window size.

**Supplementary Figure S3:** Major subfamilies of transposable element in *Pantala flavescens* genome. DNA: DNA transposon; LINE: long interspersed nuclear element; LTR: long terminal repeat; SINE: short interspersed nuclear element.

giac009_GIGA-D-21-00299

giac009_GIGA-D-21-00299_R1

giac009_Response_to_Reviewer_Comments_Revision_1

giac009_Reviewer_1_Report_Original_Submission Panagiotis Ioannidis -- 10/31/2021 Reviewed

giac009_Reviewer_1_Report_Revision_1 Panagiotis Ioannidis -- 12/10/2021 Reviewed

giac009_Reviewer_2_Report_Original_Submission Isabel Almudi -- 11/3/2021 Reviewed

giac009_Reviewer_2_Report_Revision_1 Isabel Almudi -- 11/29/2021 Reviewed

giac009_Supplemental_File

## Abbreviations

BLAST: Basic Local Alignment Search Tool; bp: base pairs; BUSCO: Benchmarking Universal Single-Copy Orthologs; BWA: Burrows-Wheeler Aligner; CCS: circular consensus sequencing; GATK: Genome Analysis Toolkit; Gb: gigabase pairs; kb: kilobase pairs; KEGG: Kyoto Encyclopedia of Genes and Genomes; Mb: megabase pairs; Mya: million years ago; NCBI: National Center for Biotechnology Information; PacBio: Pacific Biosciences; RAxML: Randomized Axelerated Maximum Likelihood; SMRT: Single Molecule, Real-Time; SNP: single-nucleotide polymorphism; SRA: Sequence Read Archive; TE: transposable element.

## Conflict of Interests

The authors declare that they have no competing interests.We declare that we do not have any commercial or associative interest that represents a conflict of interest in connection with the work submitted.

## Funding

This work was funded by Shenzhen Science and Technology Program (Grant No. KQTD20180411143628272); Fund of Key Laboratory of Shenzhen (ZDSYS20141118170111640); and The Agricultural Science and Technology Innovation Program.

## Authors’ Contributions

H.L., A.W., and D.X. collected the samples and extracted the DNA. H.L. analyzed the data and wrote the manuscript. F.J., S.W., H.W., B.Y., and H.Z. provided helpful suggestions. W.F. conceived the study, designed the experiments, and revised the manuscript. All authors read and approved the final manuscript.
