# Supplementary material for: Chromosome-level genome of the globe skimmer dragonfly (Pantala flavescens)
Source: Gigascience. 2022 Apr 4;11:giac009. doi: 10.1093/gigascience/giac009 (PMC8978299; doi:10.1093/gigascience/giac009)
Supplement: giac009_GIGA-D-21-00299 [file giac009_giga-d-21-00299.pdf]

GigaScience

Chromosome-level genome of the globe skimmer dragonfly (Pantala flavescens)

--Manuscript Draft--

Manuscript Number:	GIGA-D-21-00299
Full Title:	Chromosome-level genome of the globe skimmer dragonfly (Pantala flavescens)
Article Type:	Data Note
Funding Information:	The Agricultural Science and Technology Innovation Program — Ms. wei fan<br>Shenzhen Science and Technology Program (KQTD20180411143628272) — Ms. wei fan<br>Fund of Key Laboratory of Shenzhen (ZDSYS20141118170111640) — Ms. wei fan
Abstract:	Background: The globe skimmer dragonfly (Pantala flavescens) is a notable Odonata insect distributed in nature fields and farmlands worldwide and is commonly recognized as a natural enemy, as it preys on agricultural pests and health pests. Composing one of the sister groups of winged insects, odonatan species are key to understanding insect evolution. Findings: We present a chromosome-level reference genome of P. flavescens , which is also the first chromosome-level genome in the Palaeoptera (a subclass of insects that are unable to flex their wings over their abdomen and includes the orders Odonata and Ephemeroptera). The assembled genome size was 662 Mb, with a contig N50 of 16.2 Mb. Via Hi-C scaffolding, 648 Mb (97.6%) of contig sequences were clustered, ordered and assembled into 12 large scaffolds, each corresponding to a natural chromosome. The repetitive sequences and gene density of the X chromosome were similar to those of autosomal sequences, but the X chromosome showed a much lower degree of heterozygosity. Our analysis shows that the effective population experienced three declining events, which may have been caused by climate change and environmental pollution. Conclusions: The genome of P. flavescens is conducive for the utilization of this species in pest control and provides more information on the biology and evolution of insects.
Corresponding Author:	Wei Fan<br>Chinese Academy of Agricultural Sciences<br>CHINA
Corresponding Author Secondary Information:	
Corresponding Author's Institution:	Chinese Academy of Agricultural Sciences
Corresponding Author's Secondary Institution:	
First Author:	hangwei liu
First Author Secondary Information:	
Order of Authors:	hangwei liu
	fan jiang
	sen wang
	hengchao wang
	anqi wang
	hanbo zhao
	dong xu
	boyuan yang
	wei fan

Powered by Editorial Manager® and ProduXion Manager® from Aries Systems Corporation

Order of Authors Secondary Information:	
Additional Information:	
Question	Response
Are you submitting this manuscript to a special series or article collection?	No
**Experimental design and statistics**<br><br>Full details of the experimental design and statistical methods used should be given in the Methods section, as detailed in our Minimum Standards Reporting Checklist. Information essential to interpreting the data presented should be made available in the figure legends.<br><br>Have you included all the information requested in your manuscript?	Yes
**Resources**<br><br>A description of all resources used, including antibodies, cell lines, animals and software tools, with enough information to allow them to be uniquely identified, should be included in the Methods section. Authors are strongly encouraged to cite Research Resource Identifiers (RRIDs) for antibodies, model organisms and tools, where possible.<br><br>Have you included the information requested as detailed in our Minimum Standards Reporting Checklist?	Yes
**Availability of data and materials**<br><br>All datasets and code on which the conclusions of the paper rely must be either included in your submission or deposited in publicly available repositories (where available and ethically appropriate), referencing such data using a unique identifier in the references and in the “Availability of Data and Materials” section of your manuscript.	Yes

Have you have met the above requirement as detailed in our Minimum Standards Reporting Checklist?	

Manuscript

Click here to access/download;Manuscript;Manuscript2020923.docx

Chromosome- level genome of the globe skimmer dragonfly (*Pantala flavescens*)

Hangwei Liu, Fan Jiang, Sen Wang, Hengchao Wang, Anqi Wang, Hanbo Zhao, Dong Xu, Boyuan Yang, Wei Fan*

Guangdong Laboratory for Lingnan Modern Agriculture (Shenzhen Branch), Genome Analysis Laboratory of the Ministry of Agriculture and Rural Affairs, Agricultural Genomics Institute at Shenzhen, Chinese Academy of Agricultural Sciences, Shenzhen, Guangdong, 518120, China

*Correspondence address. Wei Fan, Agricultural Genomics Institute at Shenzhen, Chinese Academy of Agricultural Sciences, 7 Pengfai Road, Shenzhen 518120, China. E-mail: fanwei@caas.cn.

ABSTRACT

Background: The globe skimmer dragonfly (*Pantala flavescens*) is a notable Odonata insect distributed in nature fields and farmlands worldwide and is commonly recognized as a natural enemy, as it preys on agricultural pests and health pests. Composing one of the sister groups of winged insects, odonatan species are key to understanding insect evolution. **Findings:** We present a chromosome-level reference genome of *P. flavescens*, which is also the first chromosome- level genome in the Palaeoptera (a subclass of insects that are unable to flex their wings over their abdomen and includes the orders Odonata and Ephemeroptera). The assembled genome size was 662 Mb, with a contig N50 of 16.2 Mb. Via Hi-C scaffolding, 648 Mb (97.6%) of contig sequences were clustered, ordered and assembled into 12 large scaffolds, each corresponding to a natural chromosome. The repetitive sequences and gene density of the X chromosome were similar to those of autosomal sequences, but the X chromosome showed a much lower degree of heterozygosity. Our analysis shows that the effective population experienced three declining events, which may have been caused by climate change and environmental pollution. **Conclusions:** The genome of *P. flavescens* is conducive for the utilization of this species in pest control and provides

more information on the biology and evolution of insects.

Data Description

Background

The use of predatory insects has resulted in enormous economic and ecological benefits in some cases [1]. There have been many successful cases, such as the successful control of cottony cushion scale by the Vedalia ladybird beetle *Novius cardinalis* (Mulsant, 1850) in California in 1888–1889[2], *Trichogramma* spp. (Hymenoptera, Chalcidoidea, Trichogrammatidae) is the most parasitoid species used worldwide [3]. Many Odonata species are considered important natural enemies of many insect pests, such as Anopheles mosquitoes, flies, and gnats [4]. The globe skimmer dragonfly (*Pantala flavescens*), a member of the Libellulidae (Insecta: Odonata), occurs worldwide and contributes to agricultural pests and health pests [5]. Previous studies have revealed that *P. flavescens* is the most widespread species of the Odonata and is widely distributed throughout the tropics and many temperate areas, as it has a powerful capability to migrate several thousand kilometers across the globe [6-8]. Transoceanic migration of *P. flavescens* more than 10,000 km often occurs every October–December. The larval stage of *P. flavescens* is short (34–43 d)[9], which makes breeding in the ephemeral freshwater pools and floods produced by rainfall possible. However, *P. flavescens* has exhibited drastic population decreases during the last 15 years [5] due to environmental pollution and human activities, indicating that more attention needs to be paid to this species.

Odonata are diverse, numerous, commonly observed and species rich, and more than 6000 species have been described [10, 11] ; these insects have strikingly colourful bodies, giant compound eyes and an active flying ability. Odonata consists of two main suborders, Anisoptera (dragonflies) and Zygoptera (damselflies), which show significant discrepancies. Dragonflies are generally robust, and their wings spread flat at rest, while damselflies are slender to build and hold their wings over their abdomen when they at rest[10]. Odonata species date to the Carboniferous (360-290 million years ago) according to many complete and well-preserved fossil records [12]. Odonata and

Ephemeroptera (mayflies) are members of Palaeopteran insects, which is the first winged insect and the sister of Neopterans[13]. The evolution of wings in insects is a major event, as the appearance of wings has caused insects to become the largest and most abundant animal taxon on Earth[14, 15]. Powerful flight capabilities with varied wing dimorphism facilitate winged insect (Pterygota) migration, escape and mating, and more resources and habitats can be occupied by Pterygota insects.

Genomic resources for insects available in public databases are mainly focused on dipteran flies, lepidopterans and hymenopterans, most of which are sanitary or agricultural pests. They do not capture the profile of whole insects, hindering the study of insect evolution. Recent advances in circular consensus sequencing (CCS), which can generate highly accurate (99.8%), long, high-fidelity (HiFi) reads[16], combined with sophisticated assembly software such as hifiasm [17] and HiCanu[18] make it relatively easy to obtain high-quality insect genomic data. Thus, we can study Odonata from a genomic perspective, which should provide more fundamental insights across insects. To date, only four genomes of Odonata species with low continuity have been released, and a high-quality genome of Odonata species is necessary for research.

Odonata species present many pivotal characteristics, and their specific phylogenetic positions make them irreplaceable models for studying the biology and evolution of insects. Despite the attractiveness of this group for evolutionary genomic analysis, efforts have lagged behind those of other insect orders. In this study, we sequenced the genome of *P. flavescens*, as a representative Odonata species, with circular consensus sequencing and obtained a high-quality and chromosome-level genome along with an integral gene set. Our analyses identified potential genomic signatures, highlighting the general genomic features of Odonata species.

MATERIALS AND METHODS

Insect arrest and genomic sequencing

Male and female *P. flavescens* adults were collected at ShenZhen Station of the Chinese Academy of Agricultural Sciences, Guangdong Province, China. Insects were removed from the intestine to avoid bacterial contamination, cleaned using 30% ethanol and

ddH_2O, and then immersed in liquid nitrogen.

For Illumina sequencing, a short paired-end DNA library with a 400 bp insert size from female and male adult *P. flavescens* was constructed using standard Illumina protocols and sequenced on an Illumina HiSeq 2500 platform. For PacBio HiFi sequencing, two libraries with ~15 kb insert sizes were constructed from a female adult using PacBio SMRT. PacBio long reads were sequenced using 2 cells on a PacBio Sequel II system (Pacific Biosciences). A total of 831 Gb of subreads were generated with an N50 of 14.3 kb. Consensus reads (CCS reads) were generated using ccs software v.3.0.0 (https://github.com/PacificBiosciences/ccs) with the following parameters: --min-passes 0 --min-rq 0.99 --min-length 100 --max-length 50000. The total CCS read yield was 50 Gb, with a read length of 14.5 kb.

Total RNA was extracted separately from females and males and then mixed. Synthesized full-length cDNAs were then used to prepare three 20 kb SMRTbell template libraries for sequencing on a PacBio Sequel instrument.

Genome assembly and quality assessment

The PacBio reads were assembled using hifiasm (0.12-r304) with the following parameters: -l 1 -s 0.7. This resulted in 196 contigs with a total length of ~691 Mb and a contig N50 of 15.8 Mb. To filter duplicate contigs in the assembly, purge_dups (v1.2.3)[19] was used with the following parameters: -2 -a 50. This resulted in a purged primary assembly with a total length 661 Mb and a contig N50 of 16.2 Mb.

The quality of the assembly was evaluated using BUSCO v2.0[20] based on OrthoDB v9 from Insecta. Iso-seq full-length transcripts were also used to evaluate the accuracy of the genome. First, raw Iso-seq data were subjected to read quality filtering, read clustering, consensus calling and polishing using SMRT Analysis v.2.3 (https://github.com/PacificBiosciences/Bioinformatics-Training/wiki/Barcoding-with-SMRT-Analysis-2.3) and then assembled into high-quality and full-length transcripts. These full-length transcripts were then aligned to the genome using GMAP (version 2020-10-27)[21] to evaluate the structural accuracy of the assembly.

Genome scaffolding

A total of 170 Gb of Hi-C paired-end reads were generated, with a Q30 of 92.28%. After quality control, the clean reads were mapped to the genome by Bowtie2 (v2.3.4.3), and then HiC-Pro (v2.11.0) was used to generate an alignment file to detect valid alignments and filter multiple hits and singletons. Finally, LACHESIS[22] was used to cluster, order and orient the contigs.

Detection of X chromosome

Clean Illumina female and male read data were mapped to the chromosome-level genome with BWA, and the sequencing depth was calculated with SAMtools. The autosomes should have equal coverage, while the X chromosome should show approximately half coverage in males.

Genome annotation

A *de novo* repeat library was constructed with RepeatModeler (v1.0.8) (parameters: -engine ncbi-database). RepeatMasker was then used to identify TE repeats by combining the contents of the *de novo* repeat library and a TE database (Dfam 3.0, RepBase 20170127).

De novo prediction of coding genes was performed using repeat-masked genome sequences. The gene model parameters of AUGUSTUS [23] were trained using Iso-seq full-length transcripts. For homology-based prediction, the protein sequences of Odonata species were downloaded from the NCBI and UniProt databases and mapped to the genome with exonerate (version 2.4.0), and incomplete gene models were filtered and removed. Quality-controlled reads from two RNA libraries (SRR1184263 and SRR1184243) were mapped to the genome using Bowtie2, and StringTie was employed to construct gene prediction models. Iso-seq full-length transcripts were mapped to the genome with gmap. Finally, all the genes predicted with the four approaches were integrated with EVidenceModeler[24] to generate high-confidence gene sets.

To evaluate the accuracy of the gene sets, the coverage of highly conserved genes was assessed using BUSCO based on OrthoDB v9 of Insecta. For gene functional annotation, we aligned the protein sequences of genes with the KEGG, eggNOG, NR, and UniProt (SwissProt) databases in Diamond, with $1e^{-5}$ used as a cutoff, and obtained

the best hit. We also used InterProScan (v5.38-76.0) to search the InterPro database to identify motifs and domains.

Evolutionary analysis

Fifteen sequenced arthropoda species, including *Parasteatoda tepidariorum*, *Strigamia maritima*, *Daphnia pulex*, *Folsomia candida*, *Catajapyx aquilonaris*, *Ladona fulva*, *P. flavescens*, *Cloeon dipterum*, *Zootermopsis nevadensis*, *Zootermopsis nevadensis*, *Acyrthosiphon pisum*, *Drosophila melanogaster*, *Danaus plexippus*, *Tribolium castaneum* and *Apis mellifera*, were used to infer orthologous genes in OrthoFinder[25] with the default parameters. The protein sequences of single-copy genes from each species were aligned in MUSCLE (v3.8.1551)[26] and then concatenated into one supersequence. RAxML (version 8.2.12)[27] was subsequently used to construct a phylogenetic tree based on the concatenated supersequence with the GTRGAMMA model. Divergence times among species were calculated in MCMCtree (PAML package, v. 4.9)[28]. The calibration times were set according to the data in a previous paper: a minimum of 308 Mya and maximum of 366 Mya for *D. melanogaster* and *A. pisum*, a minimum of 413 Mya and maximum of 483 Mya for *D. melanogaster* and *C. aquilonaris*, a minimum of 413 Mya and maximum of 483 Mya for *D. melanogaster* and *C. aquilonaris*, and a minimum of 452 Mya and maximum of 557 Mya for *D. pulex* and *A. pisum* [13]. The phylogenetic tree and gene results were displayed and annotated using Evolview [29].

Demographic history

Raw reads were processed to obtain clean reads using fastp(0.20.0)[30]. The quality-controlled reads were mapped to the genome using BWA (version 0.7.15), with the default parameters. SAMtools (version 1.4) was used for sorting, and Picard (v.2.17.0) was used to remove duplicates. SNP calling was then performed using the GATK (4.0.4.0) HaplotypeCaller. To obtain high-quality SNPs, we initially used the GATK hard filter to remove the merged VCF data with the following options: $QD \geq 2.0$ && $FS \leq 60.0$ && $MQ \geq 40.0$ && $MQRankSum \geq -12.5$ && $ReadPosRankSum \geq -8.0$. SNPs present on the X chromosomes were excluded to avoid potential bias by sex.

Female and male data were used to estimate demographic history using SMC++[31]. We used a mutation rate of 1×10^{-9} per generation per year, and one generation per year.

Results

Chromosome-level genome assembly of *P. flavescens*

To obtain a high-quality genome, 50 Gb (80-fold) of high-fidelity (HiFi) reads (Table S1) from an adult female were generated with a read N50 length of 14.5 kb. Before genome *de novo* assembly, a genome survey based on k-mer frequency showed that the genome size was 663 Mb (Figure S1). The total length of the genome assembly produced by hifiasm was approximately 691 Mb, comprising 196 contigs with an N50 size of 15.8 Mb. This genome assembly is slightly larger than the estimated genome size, which may result from genome heterozygosity. Using purge_dups to reassign allelic contigs, a reference assembly was generated comprising 99 contigs with a total length of 662 Mb (Table 1), which was comparable to the estimated genome size. The contig N50 size of the genome assembly was 16.2 Mb, and the longest contig was 41.7 Mb. The completeness of the draft genome was evaluated via benchmarking universal single-copy orthologs (BUSCO)[20]. Of the 1658 single-copy orthologous genes in the BUSCO insecta_odb9 database, 1639 (98.8%) were identified in this draft genome, including 1609 (97.0%) complete and single-copy BUSCO genes and 30 (1.8%) complete and duplicated BUSCO genes. A total of 45,601 transcripts identified using PacBio single-molecule long-read sequencing were mapped to the genome assembly combined with gmap (version 2020-10-27)[21] , and 99.5% (45,366) were mapped successfully, with an average identity of 99.1% and an average coverage of 98.4%. These results also reflect the high accuracy of our assembly. Therefore, the genome assembly of *P. flavescens* presents is highly contiguous and has a high sequence quality.

To obtain credible Hi-C scaffolding results, LACHESIS was employed to anchor the contigs. The LACHESIS pipeline anchored and oriented 648 Mb (97.6%) of contigs to 12 pseudochromosomes (Table 1, Figure 1a, Figure S2), which

corresponded to the 12 chromosomes. Approximately 80% of the 31 unanchored contigs constituted repetitive sequences, which showed that most unanchored contigs were repeat fragments. The N50 size of this chromosome-level genome was 53 Mb, the longest of which was 79 Mb, and the shortest of which was 36 Mb.

A total of 117 Mb (17.8% of the nuclear genome) of interspersed repeats were identified in the *P. flavescens* genome (Table 1). DNA (31 Mb), LINEs (13 Mb) and LRTs (1.3 Mb) were the major types of TEs. The protein-coding genes in the reference genome were predicted by the EVidenceModeler pipeline combined with RNA-Seq, homologous gene data and *de novo* prediction (Table S2). A total of 14,974 gene models were predicted, with an average CDS length of 1498 bp and an average exon number of 7.1, of which approximately 99.74% (14,936) were assigned to 12 chromosomes. In terms of evaluating the completeness of the predicted gene sequences with the sequences of 1658 BUSCO genes from insecta_odb9, 1627 BUSCOs (98.1%) were determined to be complete, which is comparable to that of the genome. To identify proteins and their functions, the protein-coding gene sequences of *P. flavescens* were aligned to the contents of various databases (the Nr, UniProt, and KEGG databases). The results showed that 12754 (81%), 12771 (81%) and 9905 (68%) had homologous sequences in the NR, Uniprot and KEGG databases, respectively. Pfam annotation was performed via InterProScan. Among 14974 predicted proteins, 12934(87%) were classified by InterProScan. A total of 13,511 (91%) genes were annotated by the functional databases (Table S3). Compared to those of the other two Palaeopteran species, *C. dipterum* and *L. fulva*, the BUSCO value of *P. flavescens* was the highest (Figure 1b).

X chromosome identification

Sex chromosomes evolved from autosomes and play considerable roles in tissue development, mating and speciation [32-34]. A previous study confirmed that *P. flavescens* has an X0 sex determination, in which females possess two X chromosomes and males possess one chromosome [35]. The X chromosome was determined by mapping resequencing data from males and females to the genome

assembly. In males, the average depth of chr12 was almost half that of the other chromosomes, and the average depths of all chromosomes in females were similar (Figure 2a). Therefore, chr12, which has a total length of 36.2 Mb and contains 6 contigs, was designated as the X chromosome; this was shortest chromosome and was consistent with karyotype[35]. X0 sex determination has also been discovered in Orthoptera and some Hemiptera species such as aphids and psyllids [36, 37]. In aphids, the characteristics of the X chromosome are different from those of the autosomal chromosomes. The X chromosome of *A. pisum* is enriched in repetitive sequences, and the gene density is lower than that of the autosomes[38]. In *P. flavescens*, repeat sequences constitute 20.8% of the X chromosome, comparable to that of the autosomal sequences (17.6%), and the gene density is also comparable between the X chromosome sequence and autosomal sequences. The divergence characteristics of the X chromosome revealed various evolutionary events that affected the sex chromosome. The heterozygosity of the *P. flavescens* genome was estimated by heterozygous single-nucleotide polymorphisms (SNPs) using HiFi read data, and a sharp decrease in heterozygosity was noticed. The heterozygosity of the X chromosome was 0.5%, which was less than half that of the autosomes (1.3%) (Figure 2b). The evolution of sex chromosomes is poorly understood in Palaeopteran insects. Here, we present the first X chromosome sequence information in Palaeopteran insects, which may promote research on the evolution of sex chromosomes.

The population size decline

To investigate the genome evolutionary history of *P. flavescens*, gene family members were subjected to clustering analysis using *P. flavescens* and 14 other Arthropoda species, including chelicerates, myriapods, crustaceans, and hexapods[39]. From the gene family clustering results, 447 single-copy orthologs shared between *P. flavescens* and 14 other arthropoda species were used for phylogenetic construction and species divergence time estimation, representing arthropod evolution spanning more than 500 million years. We estimated that *P. flavescens* and *L. fulva,* which were closest relative to *P. flavescens,* shared a common ancestor at ~125 Ma, and divergence of *P.*

flavescens and *C. dipterum* was estimated to have occurred at ~420 Ma. The divergence between Palaeopteran and Neopteran insects occurred approximately 430 Mya (Fig. 2a). These phylogenetic data and estimated divergence time are mostly consistent with previous arthropod phylogenetic data[13, 39].

Effective population size (Ne) is considered a pivotal parameter in population genetics and has been applied in the analysis of evolutionary biology, conservation genetics and animal molecular breeding, as it measures genetic drift and inbreeding in real-world populations. A decline in population size comes with a loss of genetic diversity and an increase in inbreeding[40, 41], which is harmful for adaptation to complex environments. Global climate change has been recognized to profoundly reshape animal population demographics [42, 43]. Monitoring the changes in effective population size over time for wild species is important for understanding genetic health and evaluating the risk of extinction. Here, we estimated the size history of populations using SMC++[31], and SNPs present on the X chromosomes were excluded to avoid potential bias due to sex. Here, we identified three events in which the population declined and estimated a peak Ne that occurred approximately 100,000 years ago (Fig. 3b). The most ancient decline occurred during the Penultimate Glaciation [0.30–0.13 Ma], and afterwards, population expansion occurred. The second declination occurred at the Last Glacial Maximum (LGM; approximately 26.5–19 ka)[44] , which is the most recent period of extreme cold. Many wild species, such as pandas, buffaloes and ibis, experienced significant population declines during these two periods [45-47]. The results also revealed population declines in several thousand recent years (Figures 3b), which might be due to recent human exploitation and habitat loss. Evidence has indicated that, while global climate change has been the primary driver of population fluctuations for millions of years, human activities likely underlie recent population divergence and severe decline. Further genome resequencing of *P. flavescens* will allow more detailed insight into the effects of human activities on populations, such as secondary poisoning by pesticides, infrastructure construction, and reduction in food availability due to changes in human lifestyle.

DISCUSSION

Here, we present a 672 Mb chromosome-scale reference genome of *P. flavescens* obtained using PacBio HiFi and Hi-C data, which is the first chromosome-scale reference genome in the Palaeoptera, and we also identified sex chromosomes in the Palaeopterans for the first time. The BUSCO evaluation and RNA mapping percentage showed that the quality of the genome was high. The *P. flavescens* genome sequence and transcriptome datasets presented in this study provide the basis for the exploration of many important evolutionary, developmental and physiological views of the biology of arthropod species. We identified three events in which the population declined and estimated that peak Ne occurred approximately 100,000 years ago. An in-depth genome analysis would promote comprehensive utilization of *P. flavescens* and improve our understanding of insect evolution.

P. flavescens preys on many pests, including agricultural and sanitary pests; previous studies have demonstrated that *P. flavescens* is the most widespread odonatan species and has a strong capability to migrate several thousand kilometers across the globe[6, 8]. Therefore, this species has great potential for use in pest control projects. Moreover, the same key features of Odonata species, including their ancient phylogenetic position, strong migration capability and complex living environment, make these insects unparalleled models for studying the evolution and biology of insects. Generally, the genome of *P. flavescens* promote the utilization of this species in pest control and provides more information for research on insects.

In summary, we assembled a complete chromosome-level genome of a female *P. flavescens* insect, and a total of 14,974 genes were predicted. We also identified X chromosome, and demonstrate declination of the population size of *P. flavescens*. As sequencing technology advances, more genomes of pest insects and beneficial insects will be published, which will promote both integrated pest management and analysis of the evolution of insects.

Data Availability

All the raw sequencing data and genome data in this study have been deposited at NCBI as a BioProject under accession PRJNA763384. Genomic sequence reads have been deposited in the SRA database with Accession: SRR15902700, SRR15902700, SRR15910096, SRR15910131. Transcriptome sequence reads have been deposited in the SRA database with Accession: SRR15914636. Raw data of HiC have been deposited in the SRA database with Accession: SRR15910100. Genome assembly has been deposited at DDBJ/ENA/GenBank under the accession JAIUJI010000000.

Competing Interests

The authors declare that they have no competing interests.

Funding

Shenzhen Science and Technology Program (Grant No. KQTD20180411143628272); Fund of Key Laboratory of Shenzhen (ZDSYS20141118170111640); The Agricultural Science and Technology Innovation Program.

Authors' Contributions

H.L., A.W. and D.X. collected the samples and extracted the DNA. H.L. analyzed the data and wrote the manuscript. F.J., S.W., H.W., B.Y. and H.Z. provided helpful suggestions. W.F. conceived the study, designed the experiments, and revised the manuscript. All authors read and approved the final manuscript.

Acknowledgements

We thank Lingzhen Cao from Jiangxi Normal University, Limei He from Institute of Plant Protection, Chinese Academy of Agricultural Sciences for their assistance with sample collection.

Table 1: Major indicators of the *P. flavescens* genome

	Assembly feature	Value
	**Estimated genome size**	**663M**
**Contigs**	**Counts of contigs**	**662M**
	**Counts of contigs**	**99**
	**N50 size**	**16.2M**
**Scaffolds**	**Total size of scaffolds**	**648M**
	**Counts of scaffolds**	**12**
	**N50 size**	**53M**
**Genome annotation**	**Total gene number**	**14,974**
	**Average CDS length**	**1,498**
	**Average exon number**	**7.1**
**Repeat annotation**	**SINEs**	**35K**
	**LINEs**	**13M**
	**LTR**	**1.2M**
	**DNA**	**31M**
	**Unclassified**	**73M**

Figure legends

Figure 1. **The genome landscape of *Pantala flavescens.*** (a) Circular representation of the chromosomes. Tracks a-d represents the distribution of tandem repeats density, transposable elements (TEs) density, gene density and GC density, respectively, with densities calculated in 500 Kb windows. (b) BUSCO (Benchmarking Universal Single-Copy Orthologs) assessment of genomes of *P. flavescens* and other arthropoda species.

Figure 2. X chromosome identification. (a) Male: female sequence depth plotted in 500 bp of every chromosome. Red line represented the average sequencing depth. (b) Heterozygosity of X and autosome chromosome.

Figure 3. **Genome evolution of *P. flavescens.*** (a) Phylogenetic relationships and gene orthology of *P. flavescens* with other arthropoda species. The maximum likelihood phylogenomic tree was calculated based on 447 single-copy universal genes. (b) Demographic history of *P. flavescens* reconstructed from the reference and population resequencing genomes. Blue frame is represented the geological events.

Reference

1. Li H-S, Huang Y-H, Chen M-L, Ren Z, Qiu B-Y, De Clercq P, et al. Genomic insight into diet adaptation in the biological control agent Cryptolaemus montrouzieri. BMC Genomics. 2021;22 1 doi:10.1186/s12864-021-07442-3.
2. Pang H and Slipinski A. Revision of the Australian Coccinellidae (Coleoptera). Genus Diomus Mulsant. Part 1. Annales Zoologici. 2009;59:645-702. doi:10.3161/000345409X485008.
3. Smith S. Biological Control with Trichogramma: Advances, Successes, and Potential of Their Use. Annual review of entomology Vol 41. 1996;41:375-406. doi:10.1146/annurev.ento.41.1.375.
4. Cao LZ, Fu XW, Hu CX and Wu KM. Seasonal Migration of Pantala flavescens Across the Bohai Strait in Northern China. Environ Entomol. 2018;47 2:264-70. doi:10.1093/ee/nvy017.
5. Guo J, Fu X, Zhao S, Shen X, Wyckhuys KAG and Wu K. Long-term shifts in abundance of (migratory) crop-feeding and beneficial insect species in northeastern Asia. Journal of Pest Science. 2020;93 2:583-94. doi:10.1007/s10340-019-01191-9.
6. Artiss T. Phylogeography of a facultatively migratory dragonfly, Libellulaquadrimaculata (Odonata: Anisoptera). Hydrobiologia. 2004;515 1:225-34. doi:10.1023/B:HYDR.0000027332.57786.9d.
7. Feng H-Q, Wu K-M, Ni Y-X, Cheng D-F and Guo Y-Y. Nocturnal migration of dragonflies over the Bohai Sea in northern China. Ecological Entomology. 2006;31

5:511-20. doi:https://doi.org/10.1111/j.1365-2311.2006.00813.x.

8. Anderson RC. Do Dragonflies Migrate across the Western Indian Ocean? Journal of Tropical Ecology. 2009;25 4:347-58.

9. Suhling F, Schenk K, Padeffke T and Martens A. A field study of larval development in a dragonfly assemblage in African desert ponds (Odonata). Hydrobiologia. 2004;528 1:75-85. doi:10.1007/s10750-004-3047-8.

10. Bybee S, Cordoba-Aguilar A, Duryea MC, Futahashi R, Hansson B, Lorenzo-Carballa MO, et al. Odonata (dragonflies and damselflies) as a bridge between ecology and evolutionary genomics. Front Zool. 2016;13:46. doi:10.1186/s12983-016-0176-7.

11. Zhang Z-Q. Animal biodiversity: An outline of higher-level classification and survey of taxonomic richness (Addenda 2013). Zootaxa. 2013;3703:1-82. doi:10.11646/zootaxa.3703.1.1.

12. Ware J, May M and Kjer K. Phylogeny of the higher Libelluloidea (Anisoptera: Odonata): an exploration of the most speciose superfamily of dragonflies. Mol Phylogenet Evol. 2007;45 1:289-310. doi:10.1016/j.ympev.2007.05.027.

13. Misof B, Liu S, Meusemann K, Peters RS, Donath A, Mayer C, et al. Phylogenomics resolves the timing and pattern of insect evolution. Science. 2014;346 6210:763. doi:10.1126/science.1257570.

14. STORK NE. Insect diversity: facts, fiction and speculation*. 1988;35 4:321-37. doi:https://doi.org/10.1111/j.1095-8312.1988.tb00474.x.

15. Stork NE, McBroom J, Gely C and Hamilton AJ. New approaches narrow global species estimates for beetles, insects, and terrestrial arthropods. Proc Natl Acad Sci U

S A. 2015;112 24:7519-23. doi:10.1073/pnas.1502408112.

16. Wenger AM, Peluso P, Rowell WJ, Chang PC, Hall RJ, Concepcion GT, et al. Accurate circular consensus long-read sequencing improves variant detection and assembly of a human genome. Nat Biotechnol. 2019;37 10:1155-62. doi:10.1038/s41587-019-0217-9.

17. Cheng H, Concepcion GT, Feng X, Zhang H and Li H. Haplotype-resolved de novo assembly using phased assembly graphs with hifiasm. Nat Methods. 2021;18 2:170-5. doi:10.1038/s41592-020-01056-5.

18. Nurk S, Walenz BP, Rhie A, Vollger MR, Logsdon GA, Grothe R, et al. HiCanu: accurate assembly of segmental duplications, satellites, and allelic variants from high-fidelity long reads. Genome Res. 2020;30 9:1291-305. doi:10.1101/gr.263566.120.

19. Guan D, McCarthy SA, Wood J, Howe K, Wang Y and Durbin R. Identifying and removing haplotypic duplication in primary genome assemblies. Bioinformatics. 2020;36 9:2896-8. doi:10.1093/bioinformatics/btaa025 %J Bioinformatics.

20. Simão FA, Waterhouse RM, Ioannidis P, Kriventseva EV and Zdobnov EM. BUSCO: assessing genome assembly and annotation completeness with single-copy orthologs. Bioinformatics. 2015;31 19:3210-2. doi:10.1093/bioinformatics/btv351.

21. Wu T and Watanabe C. GMAP: A genomic mapping and alignment program for mRNA and EST sequences. Bioinformatics (Oxford, England). 2005;21:1859-75. doi:10.1093/bioinformatics/bti310.

22. Burton JN, Adey A, Patwardhan RP, Qiu R, Kitzman JO and Shendure J. Chromosome-scale scaffolding of de novo genome assemblies based on chromatin

interactions. Nature Biotechnology. 2013;31 12:1119-25. doi:10.1038/nbt.2727.

23. Stanke M, Steinkamp R, Waack S and Morgenstern B. AUGUSTUS: a web server for gene finding in eukaryotes. Nucleic Acids Res. 2004;32 Web Server issue:W309-12. doi:10.1093/nar/gkh379.

24. Haas BJ, Salzberg SL, Zhu W, Pertea M, Allen JE, Orvis J, et al. Automated eukaryotic gene structure annotation using EVidenceModeler and the Program to Assemble Spliced Alignments. Genome Biol. 2008;9 1:R7. doi:10.1186/gb-2008-9-1-r7.

25. Emms DM and Kelly S. OrthoFinder: solving fundamental biases in whole genome comparisons dramatically improves orthogroup inference accuracy. Genome Biol. 2015;16:157. doi:10.1186/s13059-015-0721-2.

26. Edgar R. MUSCLE: Multiple Sequence Alignment with High Accuracy and High Throughput. Nucleic acids research. 2004;32:1792-7. doi:10.1093/nar/gkh340.

27. Stamatakis A. RAxML version 8: a tool for phylogenetic analysis and post-analysis of large phylogenies. Bioinformatics. 2014;30 9:1312-3. doi:10.1093/bioinformatics/btu033.

28. Yang Z. PAML 4: phylogenetic analysis by maximum likelihood. Mol Biol Evol. 2007;24 8:1586-91. doi:10.1093/molbev/msm088.

29. He Z, Zhang H, Gao S, Lercher MJ, Chen W-H and Hu S. Evolview v2: an online visualization and management tool for customized and annotated phylogenetic trees. Nucleic Acids Research. 2016;44 W1:W236-W41. doi:10.1093/nar/gkw370 %J Nucleic Acids Research.

30. Chen S, Zhou Y, Chen Y and Gu J. fastp: an ultra-fast all-in-one FASTQ preprocessor.

Bioinformatics. 2018;34 17:i884-i90. doi:10.1093/bioinformatics/bty560 %J Bioinformatics.

31. Terhorst J, Kamm JA and Song YS. Robust and scalable inference of population history from hundreds of unphased whole genomes. Nat Genet. 2017;49 2:303-9. doi:10.1038/ng.3748.

32. Bachtrog D, Kirkpatrick M, Mank JE, McDaniel SF, Pires JC, Rice W, et al. Are all sex chromosomes created equal? Trends in Genetics. 2011;27 9:350-7. doi:https://doi.org/10.1016/j.tig.2011.05.005.

33. Bachtrog D, Mank JE, Peichel CL, Kirkpatrick M, Otto SP, Ashman TL, et al. Sex determination: why so many ways of doing it? PLoS Biol. 2014;12 7:e1001899. doi:10.1371/journal.pbio.1001899.

34. Rowe L, Chenoweth SF and Agrawal AF. The Genomics of Sexual Conflict. Am Nat. 2018;192 2:274-86. doi:10.1086/698198.

35. Walia GK, Kaur H and Kaur J. Karyotypic Variations in the Chromosome Complement of Pantala flavescens (Fabricius) of the Family Libellulidae (Anisoptera: Odonata). Cytologia. 2011;76 3:301-7. doi:10.1508/cytologia.76.301.

36. Li Y, Zhang B and Moran NA. The Aphid X Chromosome Is a Dangerous Place for Functionally Important Genes: Diverse Evolution of Hemipteran Genomes Based on Chromosome-Level Assemblies. Mol Biol Evol. 2020;37 8:2357-68. doi:10.1093/molbev/msaa095.

37. Castillo ER, Marti DA and Bidau CJ. Sex and Neo-Sex Chromosomes in Orthoptera: A Review*. Journal of Orthoptera Research. 2010;19 2:213-31.

doi:10.1665/034.019.0207.

38. Li Y, Park H, Smith TE, Moran NA and Singh N. Gene Family Evolution in the Pea Aphid Based on Chromosome-Level Genome Assembly. Molecular Biology and Evolution. 2019;36 10:2143-56. doi:10.1093/molbev/msz138.

39. Thomas GWC, Dohmen E, Hughes DST, Murali SC, Poelchau M, Glastad K, et al. Gene content evolution in the arthropods. Genome Biol. 2020;21 1:15. doi:10.1186/s13059-019-1925-7.

40. Bolton PE, Rollins LA, Brazill-Boast J, Maute KL, Legge S, Austin JJ, et al. Genetic diversity through time and space: diversity and demographic history from natural history specimens and serially sampled contemporary populations of the threatened Gouldian finch (Erythrura gouldiae). Conservation Genetics. 2018;19 3:737-54. doi:10.1007/s10592-018-1051-1.

41. Wang J, Santiago E and Caballero A. Prediction and estimation of effective population size. Heredity. 2016;117 4:193-206. doi:10.1038/hdy.2016.43.

42. Root TL and Schneider SH. Ecology and Climate: Research Strategies and Implications. Science. 1995;269 5222:334. doi:10.1126/science.269.5222.334.

43. Hewitt G. The genetic legacy of the Quaternary ice ages. Nature. 2000;405 6789:907-13. doi:10.1038/35016000.

44. Clark PU, Dyke AS, Shakun JD, Carlson AE, Clark J, Wohlfarth B, et al. The Last Glacial Maximum. Science. 2009;325 5941:710-4. doi:10.1126/science.1172873.

45. Zhao S, Zheng P, Dong S, Zhan X, Wu Q, Guo X, et al. Whole-genome sequencing of giant pandas provides insights into demographic history and local adaptation. Nat

Genet. 2013;45 1:67-71. doi:10.1038/ng.2494.

46. Liu Q, Ruan J, Shi D, Chen B, Qian Q, Ye G, et al. Understanding divergent domestication traits from the whole-genome sequencing of swamp- and river-buffalo populations. National Science Review. 2020;7 3:686-701. doi:10.1093/nsr/nwaa024.

47. Feng S, Fang Q, Barnett R, Li C, Han S, Kuhlwilm M, et al. The Genomic Footprints of the Fall and Recovery of the Crested Ibis. Curr Biol. 2019;29 2:340-9 e7. doi:10.1016/j.cub.2018.12.008.

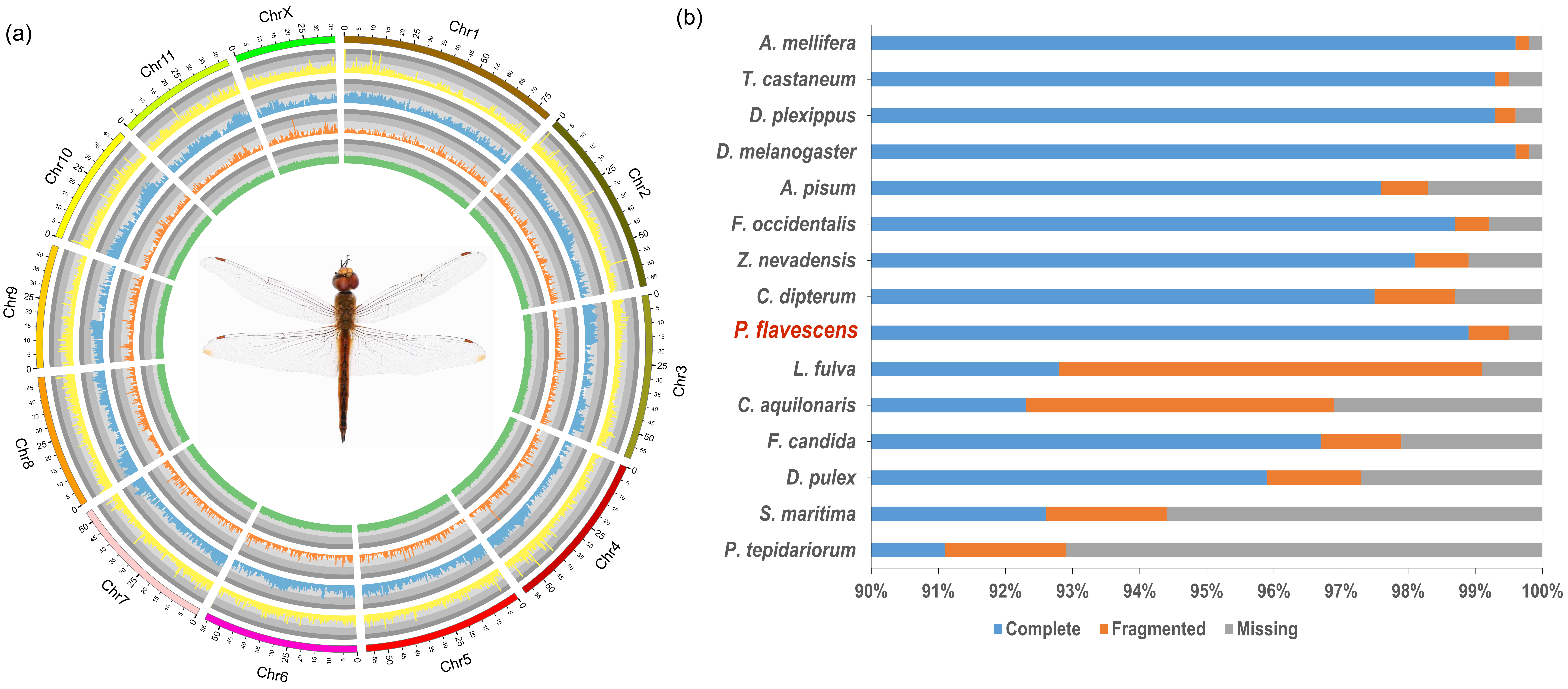
(a)
ChrX
Chr1
Chr2
Chr3
Chr4
Chr5
Chr6
Chr7
Chr8
Chr9
Chr10
Chr11
(b)
A. mellifera
T. castaneum
D. plexippus
D. melanogaster
A. pisum
F. occidentalis
Z. nevadensis
C. dipterum
P. flavescens
L. fulva
C. aquilonaris
F. candida
D. pulex
S. maritima
P. tepidariorum
90%
91%
92%
93%
94%
95%
96%
97%
98%
99%
100%
Complete
Fragmented
Missing

Click here to access/download;Figure;Figure2.pdf

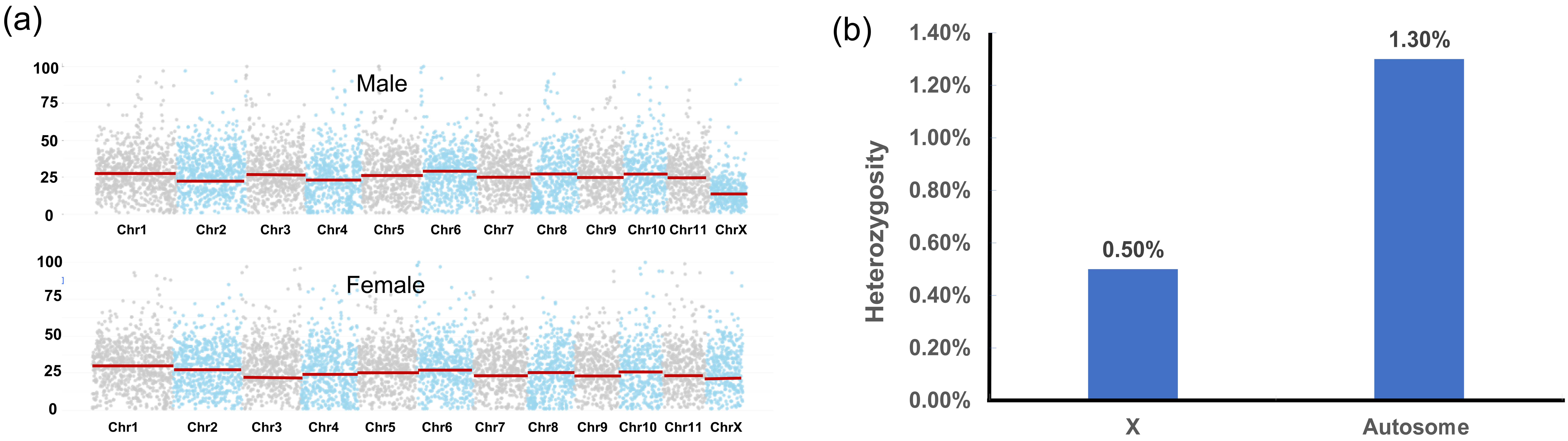

Figure3

Click here to access/download;Figure;Figure3.pdf

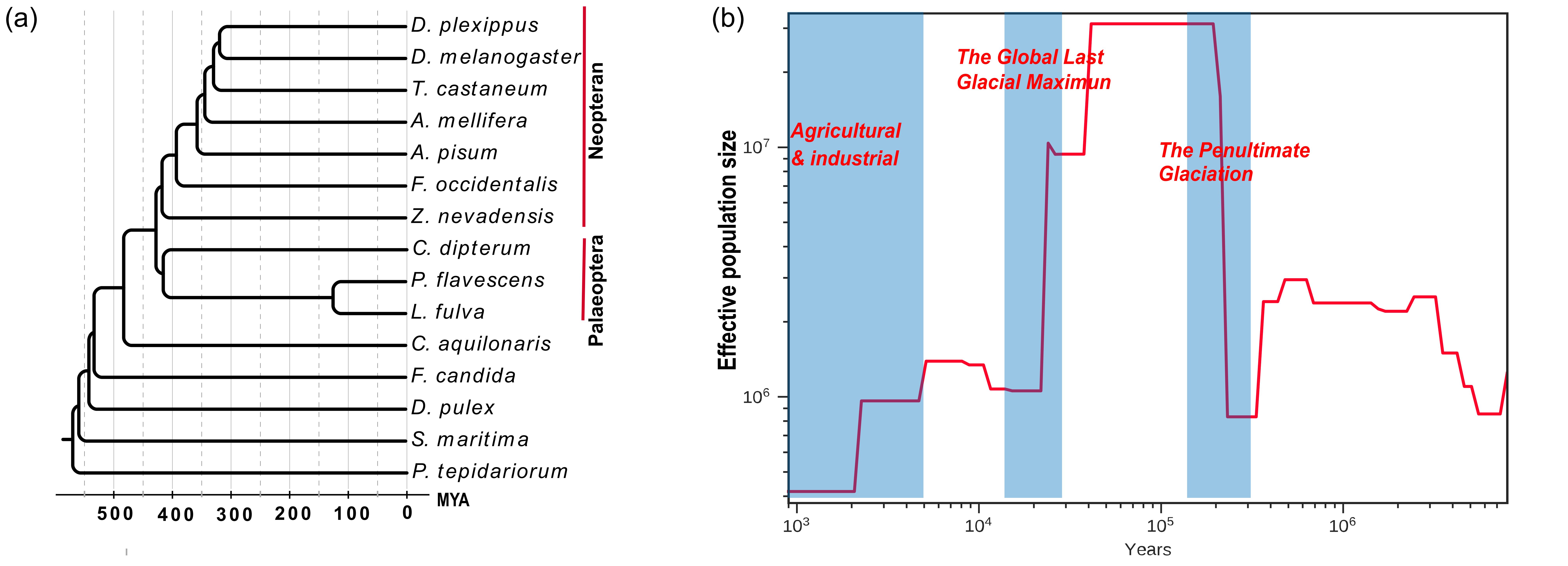

Click here to access/download
Supplementary Material
Supplementary Information.docx

Personal Cover

Click here to access/download;Personal Cover;Cover letter.docx

Dear Editor,

We are delighted to submit the manuscript entitled "Chromosome- level genome of the globe skimmer dragonfly (*Pantala flavescens*)" as a *Data Note* to *GigaScience*.

The globe skimmer dragonfly (*Pantala flavescens*) is a notable Odonata insect distributed in nature fields and farmlands worldwide and is commonly recognized as a natural enemy, as it preys on agricultural pests and health pests. Composing one of the sister groups of winged insects, odonatan species are key to understanding insect evolution. Despite the attractiveness of this group for evolutionary genomic analysis, genomic resources of Odonata insect are limited.

Here, we sequenced the genome of *P. flavescens* with circular consensus sequencing (CCS) which can generate highly accurate (99.8%), long, high-fidelity (HiFi) reads. Our analyses identified potential genomic signatures:

(1) We present a 662Mb chromosome-level reference genome of *P. flavescens*, the first chromosome- level genome in the Palaeoptera.

(2) The X chromosome was identified

(3) Our results revealed three declining events of effective population which may have been caused by climate change and environmental pollution.

We confirm that the whole manuscript has not been published or submitted for publication elsewhere. All authors declare that they have no competing interests, and approve the manuscript for submission.

We appreciate your consideration of our work and look forward to hearing from you.

Sincerely yours,

Wei Fan, Professor

E-mail: fanwei@caas.cn

Agricultural Genomics Institute at Shenzhen, Chinese Academy of Agricultural Sciences, Shenzhen 518120, China
