# Supplementary material for: Chromosome-level genome of the globe skimmer dragonfly (Pantala flavescens)
Source: Gigascience. 2022 Apr 4;11:giac009. doi: 10.1093/gigascience/giac009 (PMC8978299; doi:10.1093/gigascience/giac009)
Supplement: giac009_GIGA-D-21-00299_R1 [file giac009_giga-d-21-00299_r1.pdf]

GigaScience

Chromosome-level genome of the globe skimmer dragonfly (Pantala flavescens)

--Manuscript Draft--

Manuscript Number:	GIGA-D-21-00299R1
Full Title:	Chromosome-level genome of the globe skimmer dragonfly (Pantala flavescens)
Article Type:	Data Note
Funding Information:	Shenzhen Science and Technology Program (KQTD20180411143628272) — Ms. wei fan<br>Fund of Key Laboratory of Shenzhen (ZDSYS20141118170111640) — Ms. wei fan<br>The Agricultural Science and Technology Innovation Program — Ms. wei fan
Abstract:	Background: The globe skimmer dragonfly (Pantala flavescens) is a notable Odonata insect distributed in nature fields and farmlands worldwide and is commonly recognized as a natural enemy, as it preys on agricultural pests and health pests. Composing one of the sister groups of winged insects, odonatan species are key to understanding insect evolution. Findings: We present a chromosome-level reference genome of P. flavescens , which is also the first chromosome-level genome in the Palaeoptera (a subclass of insects that are unable to flex their wings over their abdomen and includes the orders Odonata and Ephemeroptera). The assembled genome size was 662 Mb, with a contig N50 of 16.2 Mb. Via Hi-C scaffolding, 648 Mb (97.6%) of contig sequences were clustered, ordered and assembled into 12 large scaffolds, each corresponding to a natural chromosome. The repetitive sequences and gene density of the X chromosome were similar to those of autosomal sequences, but the X chromosome showed a much lower degree of heterozygosity. Our analysis shows that the effective population experienced three declining events, which may have been caused by climate change and environmental pollution. Conclusions: The genome of P. flavescens is conducive for the utilization of this species in pest control and provides more information on the biology and evolution of insects.
Corresponding Author:	Wei Fan<br>Chinese Academy of Agricultural Sciences<br>CHINA
Corresponding Author Secondary Information:	
Corresponding Author's Institution:	Chinese Academy of Agricultural Sciences
Corresponding Author's Secondary Institution:	
First Author:	hangwei liu
First Author Secondary Information:	
Order of Authors:	hangwei liu
	fan jiang
	sen wang
	hengchao wang
	anqi wang
	hanbo zhao
	dong xu
	boyuan yang
	wei fan

Powered by Editorial Manager® and ProduXion Manager® from Aries Systems Corporation

Order of Authors Secondary Information:	
Response to Reviewers:	GIGA-D-21-00299<br>Chromosome-level genome of the globe skimmer dragonfly (Pantala flavescens)<br>hangwei liu; fan jiang; sen wang; hengchao wang; anqi wang; hanbo zhao; dong xu; boyuan yang; wei fan<br>GigaScience<br><br>Dear Dr. Fan,<br><br>Your manuscript "Chromosome-level genome of the globe skimmer dragonfly (Pantala flavescens)" (GIGA-D-21-00299) has been assessed by our reviewers. Based on these reports, and my own assessment as Editor, I am pleased to inform you that it is potentially acceptable for publication in GigaScience, once you have carried out some essential revisions suggested by our reviewers.<br><br>Their reports, together with any other comments, are below. Please also take a moment to check our website at https://www.editorialmanager.com/giga/ for any additional comments that were saved as attachments.<br><br>In addition, please register any new software application in the bio.tools and SciCrunch.org databases to receive RRID (Research Resource Identification Initiative ID) and biotoolsID identifiers, and include these in your manuscript. This will facilitate tracking, reproducibility and re-use of your tool.<br><br>Once you have made the necessary corrections, please submit a revised manuscript online at:<br><br>https://www.editorialmanager.com/giga/<br><br>If you have forgotten your username or password please use the "Send Login Details" link to get your login information. For security reasons, your password will be reset.<br><br>Please include a point-by-point within the 'Response to Reviewers' box in the submission system. Please ensure you describe additional experiments that were carried out and include a detailed rebuttal of any criticisms or requested revisions that you disagreed with. Please also ensure that your revised manuscript conforms to the journal style, which can be found in the Instructions for Authors on the journal homepage. If the data and code has been modified in the revision process please be sure to update the public versions of this too.<br><br>The due date for submitting the revised version of your article is 01 Feb 2022.<br><br>We look forward to receiving your revised manuscript soon.<br><br>Best wishes,<br><br>Hongfang Zhang<br>GigaScience<br>www.gigasciencejournal.com<br><br>Reviewer reports:<br>Reviewer #1: This paper describes the sequencing, assembly and annotation of a dragonfly (P. flavescens) genome. Very importantly, the assembly is at chromosome level, making it extremely useful for anyone who wants to run reliable comparative genomics analyses. Effectively, the authors have produced a reference palaeopteran genome. In addition to generating this very useful genomic resource, the authors have run a couple of analyses such as the study of the effective population size along evolutionary time, and a phylogenomic analysis.<br><br>I did note that the BUSCO score of the gene set is less than that of the genome assembly. The truth is that the difference is small; only 0.7%. Nevertheless, this means that the gene prediction pipeline doesn't work very well. If BUSCO was able to find 98.8% of conserved genes, then a usually more "sophisticated" gene prediction pipeline should be able to find at least the same number of genes (if not more). Quite

often these genes could be missed because they're marked as repeats by RepeatModeler. For this reason, manual inspection of the "unknown" repeats is encouraged in order to exclude "normal" genes that look like repeats (e.g. duplicated genes). For the purposes of this Data Note and since the difference is small, I believe that it might not be necessary for the authors to improve the predicted gene set. If they decide to do so, then that would be great for anyone who would use their data in the future.
Response: Thanks for the reviewer's kind suggestion, and we have refined the repeat annotation and re-run the gene annotation.

To avoid protein-coding genes being marked as repeats, we aligned the 260 repeat sequences of 'unknown' type to NR database by blastx (v2.7.1+) using 1e−5 as cutoff, and 53 of them were found to have homology with known non-TE protein-coding genes, which were filtered out of RepeatModeler de novo library. Repeatmasker was then used to find TEs based on the filtered de novo TE library. After that, we re-run the gene annotation pipeline, and 15,354 genes were annotated. This time, we used the latest BUSCO (v5.2.2) version with insecta_odb10 to assess the gene set, and showed that 98.9% genes was complete, higher than the value of genome 96.9%.

The method of genome annotation in line138 has also been updated.

Regarding the methodology and results on the effective population size, I'm not an expert and therefore I cannot judge their correctness. As for the phylogenomic analysis, it was properly done and the results obtained are correct.

Last, while I was able to have a look at the deposited raw reads (PacBio and Illumina) in SRA, I wasn't able to see the deposited assembly and gene set. I know that it is possible to generate "reviewer links" for SRA submissions, but I'm not sure if it is also the case for submitted genome assemblies and gene sets. If it is possible, could the authors generate such a link?
Response: Now the assembly has been released in NCBI (https://www.ncbi.nlm.nih.gov/genome/103548), and the gene set has also been uploaded to NCBI. These data are also available in AGIS ftp: ftp://ftp.agis.org.cn/~fanwei/Pantala_flavscens/

Some minor corrections:

- Methods -> Evolutionary analysis: In the beginning of the section, where you mention the species you used, Zootermopsis is mentioned twice.
Response: We have revised it.

- Table S1: in the last column of the table, all numbers are "Gbp", not "G". The same is true for the numbers mentioned in the last column of Table 1; they should be "Mbp" and "Kbp".
Response: We have revised it.
- Table S2: I presume that the second column refers to homology-based prediction. If true, then please change the title of the column to "Homology", because "Homo" is confusing.
Response: We have revised "Homo" to "Homology".
- Table S3: "Counts" doesn't adequately describe the numbers in this column. Maybe something like "Number of genes with significant similarity" would be more appropriate.
Response: We have revised "Counts" to "Number of genes".

Reviewer #2: In this manuscript Hangwei Liu and co-authors report the sequencing and genome assembly of the dragonfly species, Pantala flavescens.
This 662 Mb genome assembly is distributed in 12 chromosomes. Genome annotation resulted in almost 15,000 genes, corresponding to a high degree of completeness using BUSCO databases. The authors also identify the sexual chromosome (X) by comparing the ratio of sequenced reads between male and females. Finally, they performed an estimation of the demographic history and detected three events of population decline.
The work will be relevant for the fields of evolutionary biology, evolutionary genomics and researchers working in the evolution of insects. Although I am missing further analyses of different genome features that would increase the scope of the manuscript-

especially those mentioned in the introduction, such as the appearance of wings in insects or the study of gene families important for migration, insect ecology, etc. -, a chromosome grade genome of an Odonata species is of great value for the community. Therefore, I recommend the publication of this manuscript as a Data Note in GigaScience. However, I have some comments that should be addressed prior publication:

1. Since this is a Data note manuscript, a more detailed methodology would be recommended. It is not clear to me how many males or females have been used for the different sequencing protocols: for the PacBio Hifi one female was used, what about the Illumina and the RNA-seq?
Response: We have revised the sequencing protocols in method to make it clearer.

line 96: “For Illumina sequencing, a short paired-end DNA library with a 400 bp insert size from female and male adult P. flavescens” have been revised to “For Illumina sequencing, a short paired-end DNA library with a 400 bp insert size from a female adult and a male adult P. flavescens”.

line 105: We revised “Total RNA was extracted separately from females and males and then mixed” to “Total RNA was extracted from a female adult and a male adult and then mixed to generate the libraries.”

Line 124: We revised “A total of 170 Gb of Hi-C paired-end reads were generated” to “A total of 170 Gb of Hi-C paired-end reads were generated from a female adult”

- "Insects were removed from the intestine to avoid bacterial contamination,":
Is it just bacterial contamination or also contamination from prey (insects?) genomes? Please clarify.
Response: The contamination includes bacterial and prey genomes. So, we have revised line93 “Insects were removed from the intestine to avoid bacterial contamination” to “Insects were removed from the intestine to avoid contamination from bacterial and prey genomes”.

- "Total RNA was extracted separately from females and males and then mixed": Does it mean that the total RNA was mixed to generate the libraries or that libraries and sequencing was done independently for male and females and the data was merged for the subsequent analyses?
Response: Our meaning is that total RNA was mixed to generate the libraries. And to make it clearer, we revised line 105“Total RNA was extracted separately from females and males and then mixed” to “Total RNA was extracted from a female adult and a male adult and then mixed to generate the libraries.”

2. BUSCO analysis and comparisons. Perhaps a table with the percentage of completeness for the different arthropods would be clearer to visualize instead of the plot with the horizontal bars.
Response: We have changed Figure1b to a Table2.

- Also regarding the BUSCO data, it would be good if you listed the source of these numbers from other arthropods, referencing the primary articles, especially in the case of Ladona fulva (https://doi.org/10.15482/USDA.ADC/1503790.) and Cloeon dipterum (https://doi.org/10.1038/s41467-020-16284-8), since they are both respectively used as the most closely related genome or as an outgroup within the Paleoptera lineage along the entire manuscript. Actually, for the C. dipterum data, the original paper reported 96.9% complete and 1.3% fragmented whereas according to the figure 1b, C. dipterum genome has more than 97% of complete and around 1% of fragmented sets, could you explain this minor discrepancy?
Response: Thanks for the suggestion, and we have revised the reference and cited the primary articles about other genomes used in this manuscript.

The BUSCO assessments were performed by ourselves using BUSCO (5.2.2), not copied from reference papers. So, the discrepancy of C. dipterum BUSCO assessment between the original paper and this manuscript might be caused by different versions of BUSCO and insecta_odb.

	3. "Genomic resources for insects available in public databases are mainly focused on dipteran flies, lepidopterans and hymenopterans":<br>While it is true that historically available genomes belong mainly to Diptera, Lepidoptera and other holometabola, genome projects for hemimetabolous insects have been developed recently, thus acknowledging the existence of these efforts and new genomes would be desirable: see for instance crickets: Ylla et al. 2021(https://doi.org/10.1038/s42003-021-02197-9), Ephemeroptera: Almudi et al. 2020 (https://doi.org/10.1038/s41467-020-16284-8), damselfly: Ioannidis et al. 2017 (https://doi.org/10.1093/gbe/evx006), Sinella curviseta, collembola: Zhang et al. 2019 (https://doi.org/10.1093/gbe/evz013), giant collembolan: Wu et al. 2017 (https://doi.org/10.1186/s12864-017-4197-1), water strider: Armisén et al. 2018 (https://doi.org/10.1186/s12864-018-5163-2), cockroach: Harrison et al. 2018 (https://doi.org/10.1038/s41559-017-0459-1), among many others.<br>Response: Thanks for the suggestion, we have revised<br>line67 “Genomic resources for insects available in public databases are mainly focused on dipteran flies, lepidopterans and hymenopterans, most of which are sanitary or agricultural pests. They do not capture the profile of whole insects, hindering the study of insect evolution.”<br>into<br>“Genomic resources for insects available in public databases include dipteran flies, lepidopterans, hymenopterans, blattarias, and so on. However, only four genomes of Odonata species with low continuity have been released, and a high-quality genome of Odonata species is necessary for research.”.<br><br>4. The text needs some proofreading, I detected some typos or sentences that sound a bit odd:<br><br>- 4a. Page 4:<br>"… is the most parasitoid species used worldwide [3]."<br>I think that the authors probably meant:<br>"…is the parasitoid species most used worldwide"<br>Response: The sentence has been revised according to the reviewer’s kind suggestion.<br><br>- 4b. Page 5:<br>"of Palaeopteran insects, which is the first winged insect and the sister of Neopterans"<br>change to:<br>"of Palaeopteran insects, which are the first winged insects and the sister group of Neopterans "<br>Response: The sentence has been revised according to the reviewer’s kind suggestion.<br><br>- 4c. Page 5:<br>"Powerful flight capabilities with varied wing dimorphism facilitate migration, escape and mating of winged insect (Pterygota), as well as more resources and habitats can be occupied by Pterygota insects." instead of "dimorphism facilitate winged insect (Pterygota) migration, escape and mating, and more resources and habitats can be occupied by Pterygota insects."<br>Response: The sentence has been revised according to the reviewer’s kind suggestion.<br><br>- 4d. Page 5:<br>"Despite the attractiveness of this group for evolutionary genomic analysis, efforts have lagged behind for other insect orders." instead of "Despite the attractiveness of this group for evolutionary genomic analysis, efforts have lagged behind those of other insect orders."<br>Response: The sentence has been revised according to the reviewer’s kind suggestion.<br><br>- 4e. Page 8:<br>"Zootermopsis nevadensis, Zootermopsis nevadensis," appears duplicated.<br>Response: The sentence has been revised according to the reviewer’s kind suggestion.

Powered by Editorial Manager® and ProduXion Manager® from Aries Systems Corporation

	- 4f. Page 9:<br>"Therefore, the genome assembly of P. flavescens is a high-quality and highly contiguous genome." instead of "Therefore, the genome assembly of P. flavescens presents is highly contiguous and has a high sequence quality."<br>Response: The sentence has been revised according to the reviewer's kind suggestion.<br><br>5. Supplementary figure legends are missing, a brief description of the figure should be added besides the title of the figure.<br>Response: We have added figure legends and a brief description to Supplementary figures.<br><br>Please also take a moment to check our website at for any additional comments that were saved as attachments. Please note that as GigaScience has a policy of open peer review, you will be able to see the names of the reviewers.
**Additional Information:**	
**Question**	**Response**
Are you submitting this manuscript to a special series or article collection?	No
**Experimental design and statistics**<br><br>Full details of the experimental design and statistical methods used should be given in the Methods section, as detailed in our Minimum Standards Reporting Checklist. Information essential to interpreting the data presented should be made available in the figure legends.<br><br>Have you included all the information requested in your manuscript?	Yes
**Resources**<br><br>A description of all resources used, including antibodies, cell lines, animals and software tools, with enough information to allow them to be uniquely identified, should be included in the Methods section. Authors are strongly encouraged to cite Research Resource Identifiers (RRIDs) for antibodies, model organisms and tools, where possible.<br><br>Have you included the information requested as detailed in our Minimum Standards Reporting Checklist?	Yes

**Availability of data and materials**<br><br>All datasets and code on which the conclusions of the paper rely must be either included in your submission or deposited in publicly available repositories (where available and ethically appropriate), referencing such data using a unique identifier in the references and in the "Availability of Data and Materials" section of your manuscript.<br><br>Have you have met the above requirement as detailed in our Minimum Standards Reporting Checklist?	Yes

Powered by Editorial Manager® and ProduXion Manager® from Aries Systems Corporation

Manuscript

Click here to access/download;Manuscript;Manuscript-Revise-Jan 12.liuhangwei.docx

Chromosome- level genome of the globe skimmer dragonfly (*Pantala flavescens*)

Hangwei Liu, Fan Jiang, Sen Wang, Hengchao Wang, Anqi Wang, Hanbo Zhao, Dong Xu, Boyuan Yang, Wei Fan*

Guangdong Laboratory for Lingnan Modern Agriculture (Shenzhen Branch), Genome Analysis Laboratory of the Ministry of Agriculture and Rural Affairs, Agricultural Genomics Institute at Shenzhen, Chinese Academy of Agricultural Sciences, Shenzhen, Guangdong, 518120, China

*Correspondence address. Wei Fan, Agricultural Genomics Institute at Shenzhen, Chinese Academy of Agricultural Sciences, 7 Pengfai Road, Shenzhen 518120, China. E-mail: fanwei@caas.cn.

Wei Fan [0000-0001-5036-8733];

Hangwei Liu [0000-0002-4931-1307];

Fan Jiang [0000-0003-1359-0970];

Sen Wang [0000-0001-9793-4472];

Hengchao Wang [0000-0002-8754-4195];

Anqi Wang [0000-0001-9367-2524];

Hanbo Zhao [0000-0001-9371-777X];

Dong Xu [0000-0001-6180-182X];

Boyuan Yang [0000-0001-7116-8167];

ABSTRACT

Background: The globe skimmer dragonfly (*Pantala flavescens*) is a notable Odonata insect distributed in nature fields and farmlands worldwide, and it is commonly recognized as a natural enemy as it preys on agricultural pests and health pests. As one of the sister groups of winged insects, odonatan species are key to understanding the evolution of insect wings. **Findings:** We present a high-quality reference genome of *P. flavescens*, which is the first chromosome- level genome in the Palaeoptera (Odonata and Ephemeroptera). The assembled genome size was 662 Mb, with a contig

N50 of 16.2 Mb. Via Hi-C scaffolding, 648 Mb (97.6%) of contig sequences were clustered, ordered and assembled into 12 large scaffolds, each corresponding to a natural chromosome. The X chromosome was identified by sequence coverage depth. The repetitive sequences and gene density of the X chromosome are similar to those of autosomal sequences, but the X chromosome shows a much lower degree of heterozygosity. Our analysis shows that the effective population size experienced three declining events, which may have been caused by climate change and environmental pollution. **Conclusions:** The genome of *P. flavescens* provides more information on the biology and evolution of insects, and will help for the utilization of this species in pest control.

Data Description

Background

The use of predatory insects has resulted in enormous economic and ecological benefits [1]. There have been many successful cases, such as Vedalia ladybird beetle *Novius cardinalis* (Mulsant, 1850) in control of cottony cushion scale[2], and *Trichogramma* spp. used for control of Lepidoptera pests[3]. Many odonatan species are considered important natural enemies of many insect pests such as Anopheles mosquitoes, flies, and gnats [4]. The globe skimmer dragonfly *Pantala flavescens* (NCBI:txid185825), a member of the Libellulidae (Insecta: Odonata), occurs worldwide and contributes to control of agricultural pests and health pests [5]. Previous studies have revealed that *P. flavescens* is the most widespread species of the Odonata, widely distributed throughout the tropics and many temperate areas. *P. flavescens* has a powerful capability to migrate several thousand kilometers across the globe [6-8], and transoceanic migration of *P. flavescens* more than 10,000 km often occurs every October–December. However, *P. flavescens* has exhibited drastic population decreases in the last several hundred years due to environmental pollution and human activities [5].

Odonata are diverse, numerous, commonly observed and species rich, and more than 6000 species have been described [9, 10] ; these insects have strikingly colourful bodies, giant compound eyes and an active flying ability. Odonata consists of two main

suborders, Anisoptera (dragonflies) and Zygoptera (damselflies), which show significant discrepancies. Dragonflies are generally robust, and their wings spread flat at rest, while damselflies are slender and hold their wings over their abdomen at rest[9].Odonatan species date to the Carboniferous (360-290 million years ago) according to many complete and well-preserved fossil records [11]. Odonata and Ephemeroptera (mayflies) are members of Palaeopteran insects, which are the first winged insect and the sister group of Neopterans[12]. The evolution of wings in insects is a major event, as the appearance of wings has promoted insects to become the largest and most abundant animal taxon on Earth[13, 14].

Genomic resources for Odonata available in public databases are much fewer than other orders of insects such as dipteran, lepidopteran, hymenopteran, and blattaria. Only four genomes of odonatan species (*Rhinocypha anisoptera*, *Calopteryx splendens*, *Ladona fulva*, *Ischnura elegans*) with low continuity have been released[15-17], and a high-quality genome of odonatan species is necessary for insect research. Recent advances in circular consensus sequencing (CCS), which can generate highly accurate (99.8%), long, high-fidelity (HiFi) reads[18], combined with sophisticated assembly software such as Hifiasm (Hifiasm, RRID:SCR_021069) [19] and HiCanu[20] provide a promising way to generate high-quality reference genome sequence. In this study, we sequenced the genome of *P. flavescens*, as a representative odonatan species, with HiFi technology, and obtained a chromosome-level genome assembly, along with an integral comprehensive gene set. The high-quality genomic data enables the identification and analysis of the sex chromosome, and the inferring of evolution and demographic history.

MATERIALS AND METHODS

Insect arrest and genomic sequencing

Male and female *P. flavescens* adults were collected at ShenZhen Station of the Chinese Academy of Agricultural Sciences, Guangdong Province, China. Insects were removed from the intestine to avoid contamination from bacterial and prey genomes, cleaned

using 30% ethanol and ddH_2O, and then immersed in liquid nitrogen.

For Illumina sequencing, a short paired-end DNA library with a 400 bp insert size from a female adult and a male adult *P. flavescens* was constructed using standard Illumina protocols and sequenced on an Illumina HiSeq 2500 platform (Illumina HiSeq 2500 System, RRID:SCR_016383). For PacBio HiFi sequencing, two libraries with ~15 kb insert sizes were constructed from a female adult using PacBio SMRT. PacBio long reads were sequenced using 2 cells on a PacBio Sequel II system (PacBio Sequel II System, RRID:SCR_017990). A total of 831 Gb of subreads were generated with an N50 of 14.3 kb. Consensus reads (CCS reads) were generated using ccs software v.3.0.0 [21] with the following parameters: --min-passes 0 --min-rq 0.99 --min-length 100 --max-length 50000. The total CCS read yield was 50 Gb, with a read length of 14.5 kb.

Total RNA was extracted from a female adult and a male adult and then mixed to generate the libraries. Synthesized full-length cDNAs were then used to prepare three 20 kb SMRTbell template libraries for sequencing on a PacBio Sequel instrument.

Genome assembly and quality assessment

The PacBio reads were assembled using Hifiasm (0.12-r304) with the following parameters: -l 1 -s 0.7. This resulted in 196 contigs with a total length of ~691 Mb and a contig N50 of 15.8 Mb. To filter duplicate contigs in the assembly, purge_dups (v1.2.3) (purge dups, RRID:SCR_021173) [21] was used with the following parameters: -2 -a 50. This resulted in a purged primary assembly with a total length 662 Mb and a contig N50 of 16.2 Mb.

The quality of the assembly was evaluated using BUSCO v5.2.2 (BUSCO, RRID:SCR_015008) [22] based on OrthoDB v10 of Insecta (OrthoDB, RRID:SCR_011980). Iso-seq full-length transcripts were also used to evaluate the accuracy of the genome. First, raw Iso-seq data were subjected to read quality filtering, read clustering, consensus calling and polishing using SMRT Analysis v.2.3 (SMRT-Analysis, RRID:SCR_002942) [22] and then assembled into high-quality and full-length transcripts. These full-length transcripts were then aligned to the genome using GMAP (version 2020-10-27) (GMAP, RRID:SCR_008992) [23] to evaluate the

structural accuracy of the assembly.

Genome scaffolding

A total of 170 Gb of Hi-C paired-end reads were generated from a female adult, with a Q30 of 92.28%. After quality control, the clean reads were mapped to the genome by Bowtie2 (v2.3.4.3) (Bowtie 2, RRID:SCR_016368), and then HiC-Pro (v2.11.0) (HiC-Pro, RRID:SCR_017643) was used to generate an alignment file to detect valid alignments and filter multiple hits and singletons. Finally, LACHESIS (LACHESIS, RRID:SCR_017644) [24] was used to cluster, order and orient the contigs.

Detection of X chromosome

Clean Illumina female and male read data were mapped to the chromosome-level genome with BWA, and the sequencing depth was calculated with SAMtools (SAMTOOLS, RRID:SCR_002105). The autosomes should have equal coverage, while the X chromosome should show approximately half coverage in males.

Genome annotation

A *de novo* repeat library was constructed with RepeatModeler (v1.0.8) (RepeatModeler, RRID:SCR_015027) (parameters: -engine ncbi-database). RepeatMasker (RepeatMasker, RRID:SCR_012954) was then used to identify TE repeats by combining the contents of the *de novo* repeat library and a TE database (Dfam 3.0, RRID:SCR_021168 and RepBase 20170127, RRID:SCR_021169). To avoid protein-coding genes being marked as repeats, we aligned the 260 repeat sequences of ‘unknown’ type to NR database by blastx (v2.7.1+) (BLASTX, RRID:SCR_001653) using $1e^{-5}$ as cutoff, and 53 of them were found to have homology with known non-TE protein-coding genes, which were filtered out of RepeatModeler *de novo* library. Repeatmasker was then used to find TEs based on the filtered *de novo* TE library.

De novo prediction of coding genes was performed using repeat-masked genome sequences. The gene model parameters of AUGUSTUS (Augustus, RRID:SCR_008417) [25] were trained using Iso-seq full-length transcripts. For homology-based prediction, the protein sequences of odonatan species were

downloaded from the NCBI and UniProt (UniProt, RRID:SCR_002380) databases and mapped to the genome with exonerate (version 2.4.0) (Exonerate, RRID:SCR_016088), and incomplete gene models were filtered and removed. Quality-controlled reads from two RNA libraries (SRR1184263 and SRR1184243) were mapped to the genome using Bowtie2, and StringTie (StringTie, RRID:SCR_016323) was employed to construct gene prediction models. Iso-seq full-length transcripts were mapped to the genome with GAMP. Finally, all the genes predicted with the four approaches were integrated with EVidenceModeler (EVidenceModeler, RRID:SCR_014659) [26] to generate high-confidence gene sets (Table S2).

To evaluate the accuracy of the gene sets, the coverage of highly conserved genes was assessed using BUSCO based on OrthoDB v10 of Insecta. For gene functional annotation, we aligned the protein sequences of genes with the KEGG (KEGG, RRID:SCR_012773), eggNOG (eggNOG, RRID:SCR_002456), NR, and UniProt (SwissProt) databases in Diamond, with $1e^{-5}$ used as a cutoff, and obtained the best hit. We also used InterProScan (v5.38-76.0) (InterProScan, RRID:SCR_005829) to search the InterPro (InterPro, RRID:SCR_006695) database to identify motifs and domains.

Evolutionary analysis

Fifteen sequenced arthropoda species, including *Parasteatoda tepidariorum*[27], *Strigamia maritima* [28], *Daphnia pulex* [29], *Folsomia candida* [30], *Catajapyx aquilonaris*, *L. fulva*[16], *P. flavescens*, *Cloeon dipterum* [31], *Zootermopsis nevadensis*[32], *Acyrthosiphon pisum*[33], *Drosophila melanogaster*[34], *Danaus plexippus*[35], *Tribolium castaneum*[36] and *Apis mellifera* [37], were used to infer orthologous genes in OrthoFinder (OrthoFinder, RRID:SCR_017118) [38] with the default parameters. The protein sequences of single-copy genes from each species were aligned in MUSCLE (v3.8.1551) (MUSCLE, RRID:SCR_011812) [39] and then concatenated into one supersequence. RAxML (version 8.2.12) (RAxML, RRID:SCR_006086) [40] was subsequently used to construct a phylogenetic tree based on the concatenated supersequence with the PROTGAMMALGX model. Divergence times among species were calculated in MCMCtree (PAML package, v. 4.9) (PAML,

RRID:SCR_014932) [41]. The calibration times were set according to the data in a previous paper: a minimum of 308 Mya and maximum of 366 Mya for *D. melanogaster* and *A. pisum*, a minimum of 413 Mya and maximum of 483 Mya for *D. melanogaster* and *C. aquilonaris*, a minimum of 413 Mya and maximum of 483 Mya for *D. melanogaster* and *C. aquilonaris*, and a minimum of 452 Mya and maximum of 557 Mya for *D. pulex* and *A. pisum* [12]. The phylogenetic tree and gene results were displayed and annotated using Evolview [42].

Demographic history

Raw reads were processed to obtain clean reads using fastp (0.20.0) (fastp, RRID:SCR_016962) [43]. The quality-controlled reads were mapped to the genome using BWA (version 0.7.15) (BWA, RRID:SCR_010910), with the default parameters. SAMtools (version 1.4) was used for sorting, and Picard (v.2.17.0) (Picard, RRID:SCR_006525) was used to remove duplicates. SNP calling was then performed using the GATK (4.0.4.0) (GATK, RRID:SCR_001876) HaplotypeCaller. To obtain high-quality SNPs, we initially used the GATK hard filter to remove the merged VCF data with the following options: QD ≥ 2.0 && FS ≤ 60.0 && MQ ≥ 40.0 && MQRankSum ≥ −12.5 && ReadPosRankSum ≥ −8.0. SNPs present on the X chromosomes were excluded to avoid potential bias by sex. Female and male data were used to estimate demographic history using SMC++[44]. We used a mutation rate of 1×10^{-9} per generation per year, and one generation per year.

Results

Chromosome-level genome assembly of *P. flavescens*

To obtain a high-quality genome, 50 Gb (80-fold) of high-fidelity (HiFi) reads (Table S1) from an adult female were generated with a read N50 length of 14.5 kb. Before genome *de novo* assembly, a genome survey based on k-mer frequency showed that the genome size is 663 Mb (Figure S1). The total length of the genome assembly produced by Hifiasm is approximately 691 Mb, comprising 196 contigs with an N50 size of 15.8 Mb. This genome assembly is slightly larger than the estimated genome size, which may result from genome heterozygosity. Using purge_dups to reassign

allelic contigs, a reference assembly was generated comprising 99 contigs with a total length of 662 Mb (Table 1), which is comparable to the estimated genome size. The contig N50 size of the genome assembly is 16.2 Mb, and the longest contig is 41.7 Mb. The completeness of the draft genome was evaluated via benchmarking universal single-copy orthologs (BUSCO) [22]. Of the 1,367 single-copy orthologous genes in the BUSCO insecta_odb10 database, 1,325 (96.9%) were identified in this draft genome, including 1,280 (93.6%) complete and single-copy BUSCO genes and 45 (3.3%) complete and duplicated BUSCO genes. A total of 45,601 transcripts produced using PacBio single-molecule long-read sequencing were mapped to the genome assembly with GAMP (version 2020-10-27)[23] , and 99.5% (45,366) were mapped successfully with an average identity of 99.1% and an average coverage of 98.4%. These results also reflect the high accuracy of our assembly.

The LACHESIS pipeline was employed to anchor and orient 648 Mb (97.6%) of contigs to 12 pseudochromosomes (Table 1, Figure 1, Figure S2), which corresponded to the 12 chromosomes. The N50 size of this chromosome-level genome was 53 Mb, with the longest 79 Mb and the shortest 36 Mb. Approximately 80% of the 31 unanchored contigs constituted repetitive sequences, indicating that most unanchored contigs were repeat fragments.

A total of 117 Mb (17.8% of the nuclear genome) of interspersed repeats were identified in the *P. flavescens* genome (Table 1). Among them, DNA (31 Mb), LINEs (13 Mb) and LRTs (1.3 Mb) were the major types of TEs. A total of 15,354 protein-coding gene models were predicted by EVidenceModeler (Table S2), with an average CDS length of 1,528 bp and an average exon number of 7.1, comparable to that of other published insects. In terms of evaluating the completeness of the predicted gene sequences with the sequences of 1,367 BUSCO genes from insecta_odb10, 1,352 BUSCOs (98.9%) were determined to be complete, which is better to that of the genome (96.9%). Compared to those of the other two Palaeopteran species, *C. dipterum* and *L. fulva*, the BUSCO complete ratio of *P. flavescens* is the highest (Table2). For functional annotation, 12,995 (85%), 12,417 (81%) and 10,346 (67%) genes have homologous sequences in the NR, Uniprot and KEGG databases,

respectively. In addition, 13,240(86%) genes were annotated by InterProScan. In summary, 14,024 (91%) genes were annotated by at least one functional databases or methods (Table S3).

X chromosome identification

Sex chromosomes evolved from autosomes and play important roles in tissue development, mating and speciation [45-47]. A previous study showed that *P. flavescens* has an X0 sex determination, in which females possess two X chromosomes and males possess one X chromosome [48]. The X chromosome was determined by mapping resequencing data from males and females to the genome assembly. In males, the average depth of chr12 was almost half that of the other chromosomes, and the average depths of all chromosomes in females were similar (Figure 2a). Therefore, chr12, which has a total length of 36.2 Mb and contains 6 contigs, was designated as the X chromosome. This is the shortest chromosome and is consistent with karyotype[48]. X0 sex determination has also been discovered in Orthoptera and some Hemiptera species such as aphids and psyllids [49, 50]. In aphids, the characteristics of the X chromosome are different from those of the autosomal chromosomes. The X chromosome of *A. pisum* (aphids) is enriched in repetitive sequences, and the gene density is lower than that of the autosomes[51]. However, in *P. flavescens*, repeat sequences constitute 20.8% of the X chromosome, comparable to that of the autosomal sequences (17.6%), and the gene density is also comparable between the X chromosome and autosomals.

The heterozygosity of the *P. flavescens* genome was estimated by heterozygous single-nucleotide polymorphisms (SNPs), and a sharp decrease in heterozygosity was noticed. The heterozygosity of the X chromosome is 0.5%, which is less than half that of the autosomes (1.3%) (Figure 2b). The evolution of sex chromosomes is poorly understood in Palaeopteran insects. Here, we present the first X chromosome sequence information in Palaeopteran insects, which may promote research on the evolution of sex chromosomes.

The population size decline

To investigate the genome evolutionary history of *P. flavescens*, gene family members were subjected to clustering analysis using *P. flavescens* and 14 other arthropoda species, including chelicerates, myriapods, crustaceans, and hexapods[16]. From the gene family clustering results, 447 single-copy orthologs shared between *P. flavescens* and 14 other arthropoda species were used for phylogenetic construction and species divergence time estimation, representing arthropod evolution spanning more than 500 million years. We estimated that *P. flavescens* and *L. fulva* shared a common ancestor at ~125 Ma, and divergence of *P. flavescens* and *C. dipterum* was estimated to have occurred at ~420 Ma (Fig. 2a). Our phylogenetic tree and estimated divergence time are mostly consistent with previous arthropod phylogenetic studies[12, 16].

Effective population size (Ne) is considered a pivotal parameter in population genetics and has been applied in the analysis of evolutionary biology, conservation genetics and animal molecular breeding, as it measures genetic drift and inbreeding in real-world populations. A decline in population size comes with a loss of genetic diversity and an increase in inbreeding[52, 53], which is harmful for adaptation to complex environments. Global climate change has been recognized to profoundly reshape animal population demographics [17, 54]. Monitoring the changes in effective population size over time for wild species is important for understanding genetic health and evaluating the risk of extinction. Here, we estimated the history of population sizes using SMC++[44], and identified three events in which the population declined severely (Fig. 3b). The most ancient decline occurred during the Penultimate Glaciation [0.30–0.13 Ma], and afterwards, population expansion occurred. The second declination occurred at the Last Glacial Maximum (LGM; approximately 26.5–19 ka)[55] , which is the most recent period of extreme cold. Many wild species, such as pandas, buffaloes and ibis, experienced significant population declines during these two periods [56-58]. The results also revealed population declines in the last several thousand years (Figures 3b), which might be due to recent human exploitation and habitat loss. Evidence has indicated that, while global climate change has been the primary driver of population fluctuations for millions of years, human activities likely underlie recent population divergence and

severe decline. Further genome resequencing of *P. flavescens* will provide more detailed insights into the demographic history.

DISCUSSION

Here, we present a 662 Mb chromosome-scale reference genome of *P. flavescens* obtained using PacBio HiFi and Hi-C data, which is the first chromosome-scale reference genome in the Palaeoptera, and we also identified sex chromosomes in the Palaeopterans for the first time. The high BUSCO complete ratio and RNA mapping percentage confirmed the high quality of the reference genome assembly. Our analysis showed three events in which the population declined severely. The key features of odonatan species, including their ancient phylogenetic position, strong migration capability and complex living environment, make *P. flavescens* to be a potential model of insect species. The genome and gene data of *P. flavescens* would facilitate the exploration of many important evolutionary, developmental and physiological studies on insects. Furthermore, *P. flavescens* preys on many agricultural and sanitary pests, this species has great potential for use in pest control. Our data and results will also help the development of pest management technologies.

Data Availability

All the raw sequencing data and genome data in this study have been deposited at NCBI as a BioProject under accession PRJNA763384. Genomic sequence reads have been deposited in the SRA database with Accession: SRR15902700, SRR15902700, SRR15910096, SRR15910131. Transcriptome sequence reads have been deposited in the SRA database with Accession: SRR15914636. Raw data of Hi-C have been deposited in the SRA database with Accession: SRR15910100. Genome assembly has been deposited at DDBJ/ENA/GenBank under the accession JAIUJI010000000. Supporting data and materials are available in the *GigaDB* database [61].

Competing Interests

The authors declare that they have no competing interests.

Funding

Shenzhen Science and Technology Program (Grant No. KQTD20180411143628272); Fund of Key Laboratory of Shenzhen (ZDSYS20141118170111640); The Agricultural Science and Technology Innovation Program.

Authors' Contributions

H.L., A.W. and D.X. collected the samples and extracted the DNA. H.L. analyzed the data and wrote the manuscript. F.J., S.W., H.W., B.Y. and H.Z. provided helpful suggestions. W.F. conceived the study, designed the experiments, and revised the manuscript. All authors read and approved the final manuscript.

Acknowledgements

We thank Lingzhen Cao from Jiangxi Normal University, Limei He from Institute of Plant Protection, Chinese Academy of Agricultural Sciences for their assistance with sample collection.

Table 1: Major indicators of the *P. flavescens* genome

	Assembly feature	Value
	**Estimated genome size**	**663Mbp**
**Contigs**	**Counts of contigs**	**662Mbp**
	**Counts of contigs**	**99**
	**N50 size**	**16.2Mbp**
**Scaffolds**	**Total size of scaffolds**	**648Mbp**
	**Counts of scaffolds**	**12**
	**N50 size**	**53Mbp**
**Genome annotation**	**Total gene number**	**15,354**
	**Average CDS length**	**1,528**
	**Average exon number**	**7.1**
**Repeat annotation**	**SINEs**	**35Kbp**
	**LINEs**	**13Mbp**
	**LTR**	**1.2Mbp**
	**DNA**	**31Mbp**
	**Unclassified**	**73Mbp**

Table 2: BUSCO assessment of gene sets of *P. flavescens* and other insecta species.

Species	C	S	D	F	M
A. mellifera	99.1%	61.4%	37.7%	0.2%	0.7%
T. castaneum	99.0%	98.6%	0.4%	0.3%	0.7%
D. plexippus	99.5%	97.7%	1.8%	0.2%	0.3%
D. melanogaster	99.2%	98.3%	0.9%	0.6%	0.2%
A. pisum	95.7%	89.6%	6.1%	1.0%	3.3%
F. occidentalis	98.8%	97.1%	1.7%	0.7%	0.5%
Z. nevadensis	98.0%	97.4%	0.6%	0.6%	0.8%
C. dipterum	95.2%	91.8%	3.4%	1.0%	3.8%
P. flavescens	**98.9%**	**95.5%**	**3.4%**	**0.0%**	**1.1%**
L. fulva	81.7%	79.7%	2.0%	13.0%	5.3%
C. aquilonaris	85.0%	83.6%	1.4%	6.7%	8.3%

Note: Complete BUSCOs (C), Complete and single-copy BUSCOs (S), Complete and duplicated BUSCOs (D), Fragmented BUSCOs (F), Missing BUSCOs (M).

Figure legends

Figure 1. The genome landscape of *Pantala flavescens*. Circular representation of the

chromosomes. Tracks a-d represents the distribution of tandem repeats density, transposable elements (TEs) density, gene density and GC density, respectively, with densities calculated in 500 Kb windows.

Figure 2. X chromosome identification. (a) Male and female sequence depth plotted in 500 bp of every chromosome. Red line represented the average sequencing depth. (b) Heterozygosity of X and autosome chromosome.

Figure 3. Genome evolution of *P. flavescens.* (a) Phylogenetic relationships and gene orthology of *P. flavescens* with other arthropoda species. The maximum likelihood phylogenomic tree was calculated based on 447 single-copy universal genes. (b) Demographic history of *P. flavescens* reconstructed from two adult resequencing data. Blue frame represents the geological events.

Reference

1. Li H-S, Huang Y-H, Chen M-L, Ren Z, Qiu B-Y, De Clercq P, et al. Genomic insight into diet adaptation in the biological control agent Cryptolaemus montrouzieri. BMC Genomics. 2021;22 1 doi:10.1186/s12864-021-07442-3.
2. Pang H and Slipinski A. Revision of the Australian Coccinellidae (Coleoptera). Genus Diomus Mulsant. Part 1. Annales Zoologici. 2009;59:645-702. doi:10.3161/000345409X485008.
3. Smith S. Biological Control with Trichogramma: Advances, Successes, and Potential of Their Use. Annual review of entomology Vol 41. 1996;41:375-406. doi:10.1146/annurev.ento.41.1.375.
4. Cao LZ, Fu XW, Hu CX and Wu KM. Seasonal Migration of Pantala flavescens Across the Bohai Strait in Northern China. Environ Entomol. 2018;47 2:264-70. doi:10.1093/ee/nvy017.

5. Guo J, Fu X, Zhao S, Shen X, Wyckhuys KAG and Wu K. Long-term shifts in abundance of (migratory) crop-feeding and beneficial insect species in northeastern Asia. Journal of Pest Science. 2020;93 2:583-94. doi:10.1007/s10340-019-01191-9.

6. Artiss T. Phylogeography of a facultatively migratory dragonfly, Libellulaquadrimaculata (Odonata: Anisoptera). Hydrobiologia. 2004;515 1:225-34. doi:10.1023/B:HYDR.0000027332.57786.9d.

7. Feng H-Q, Wu K-M, Ni Y-X, Cheng D-F and Guo Y-Y. Nocturnal migration of dragonflies over the Bohai Sea in northern China. Ecological Entomology. 2006;31 5:511-20. doi:https://doi.org/10.1111/j.1365-2311.2006.00813.x.

8. Anderson RC. Do Dragonflies Migrate across the Western Indian Ocean? Journal of Tropical Ecology. 2009;25 4:347-58.

9. Bybee S, Cordoba-Aguilar A, Duryea MC, Futahashi R, Hansson B, Lorenzo-Carballa MO, et al. Odonata (dragonflies and damselflies) as a bridge between ecology and evolutionary genomics. Front Zool. 2016;13:46. doi:10.1186/s12983-016-0176-7.

10. Zhang Z-Q. Animal biodiversity: An outline of higher-level classification and survey of taxonomic richness (Addenda 2013). Zootaxa. 2013;3703:1-82. doi:10.11646/zootaxa.3703.1.1.

11. Ware J, May M and Kjer K. Phylogeny of the higher Libelluloidea (Anisoptera: Odonata): an exploration of the most speciose superfamily of dragonflies. Mol Phylogenet Evol. 2007;45 1:289-310. doi:10.1016/j.ympev.2007.05.027.

12. Misof B, Liu S, Meusemann K, Peters RS, Donath A, Mayer C, et al. Phylogenomics resolves the timing and pattern of insect evolution. Science. 2014;346 6210:763.

doi:10.1126/science.1257570.

13. STORK NE. Insect diversity: facts, fiction and speculation*. 1988;35 4:321-37. doi:https://doi.org/10.1111/j.1095-8312.1988.tb00474.x.

14. Stork NE, McBroom J, Gely C and Hamilton AJ. New approaches narrow global species estimates for beetles, insects, and terrestrial arthropods. Proc Natl Acad Sci U S A. 2015;112 24:7519-23. doi:10.1073/pnas.1502408112.

15. Chauhan P, Swaegers J, Sanchez-Guillen RA, Svensson EI, Wellenreuther M and Hansson B. Genome assembly, sex-biased gene expression and dosage compensation in the damselfly Ischnura elegans. Genomics. 2021;113 4:1828-37. doi:10.1016/j.ygeno.2021.04.003.

16. Thomas GWC, Dohmen E, Hughes DST, Murali SC, Poelchau M, Glastad K, et al. Gene content evolution in the arthropods. Genome Biol. 2020;21 1:15. doi:10.1186/s13059-019-1925-7.

17. Root TL and Schneider SH. Ecology and Climate: Research Strategies and Implications. Science. 1995;269 5222:334. doi:10.1126/science.269.5222.334.

18. Wenger AM, Peluso P, Rowell WJ, Chang PC, Hall RJ, Concepcion GT, et al. Accurate circular consensus long-read sequencing improves variant detection and assembly of a human genome. Nat Biotechnol. 2019;37 10:1155-62. doi:10.1038/s41587-019-0217-9.

19. Cheng H, Concepcion GT, Feng X, Zhang H and Li H. Haplotype-resolved de novo assembly using phased assembly graphs with hifiasm. Nat Methods. 2021;18 2:170-5. doi:10.1038/s41592-020-01056-5.

20. Nurk S, Walenz BP, Rhie A, Vollger MR, Logsdon GA, Grothe R, et al. HiCanu: accurate assembly of segmental duplications, satellites, and allelic variants from high-fidelity long reads. Genome Res. 2020;30 9:1291-305. doi:10.1101/gr.263566.120.

21. Guan D, McCarthy SA, Wood J, Howe K, Wang Y and Durbin R. Identifying and removing haplotypic duplication in primary genome assemblies. Bioinformatics. 2020;36 9:2896-8. doi:10.1093/bioinformatics/btaa025 %J Bioinformatics.

22. Simão FA, Waterhouse RM, Ioannidis P, Kriventseva EV and Zdobnov EM. BUSCO: assessing genome assembly and annotation completeness with single-copy orthologs. Bioinformatics. 2015;31 19:3210-2. doi:10.1093/bioinformatics/btv351.

23. Wu T and Watanabe C. GMAP: A genomic mapping and alignment program for mRNA and EST sequences. Bioinformatics (Oxford, England). 2005;21:1859-75. doi:10.1093/bioinformatics/bti310.

24. Burton JN, Adey A, Patwardhan RP, Qiu R, Kitzman JO and Shendure J. Chromosome-scale scaffolding of de novo genome assemblies based on chromatin interactions. Nature Biotechnology. 2013;31 12:1119-25. doi:10.1038/nbt.2727.

25. Stanke M, Steinkamp R, Waack S and Morgenstern B. AUGUSTUS: a web server for gene finding in eukaryotes. Nucleic Acids Res. 2004;32 Web Server issue:W309-12. doi:10.1093/nar/gkh379.

26. Haas BJ, Salzberg SL, Zhu W, Pertea M, Allen JE, Orvis J, et al. Automated eukaryotic gene structure annotation using EVidenceModeler and the Program to Assemble Spliced Alignments. Genome Biol. 2008;9 1:R7. doi:10.1186/gb-2008-9-1-r7.

27. Schwager EE, Sharma PP, Clarke T, Leite DJ, Wierschin T, Pechmann M, et al. The

house spider genome reveals an ancient whole-genome duplication during arachnid evolution. BMC Biology. 2017;15 1:62. doi:10.1186/s12915-017-0399-x.

28. Chipman AD, Ferrier DE, Brena C, Qu J, Hughes DS, Schröder R, et al. The first myriapod genome sequence reveals conservative arthropod gene content and genome organisation in the centipede Strigamia maritima. PLoS biology. 2014;12 11:e1002005. doi:10.1371/journal.pbio.1002005.

29. Colbourne JK, Pfrender ME, Gilbert D, Thomas WK, Tucker A, Oakley TH, et al. The ecoresponsive genome of Daphnia pulex. Science. 2011;331 6017:555-61. doi:10.1126/science.1197761.

30. Faddeeva-Vakhrusheva A, Kraaijeveld K, Derks MFL, Anvar SY, Agamennone V, Suring W, et al. Coping with living in the soil: the genome of the parthenogenetic springtail Folsomia candida. BMC Genomics. 2017;18 1:493. doi:10.1186/s12864-017-3852-x.

31. Almudi I, Vizueta J, Wyatt CDR, de Mendoza A, Marletaz F, Firbas PN, et al. Genomic adaptations to aquatic and aerial life in mayflies and the origin of insect wings. Nat Commun. 2020;11 1:2631. doi:10.1038/s41467-020-16284-8.

32. Terrapon N, Li C, Robertson HM, Ji L, Meng X, Booth W, et al. Molecular traces of alternative social organization in a termite genome. Nature Communications. 2014;5 1:3636. doi:10.1038/ncomms4636.

33. Li Y, Park H, Smith TE and Moran NA. Gene Family Evolution in the Pea Aphid Based on Chromosome-Level Genome Assembly. Mol Biol Evol. 2019;36 10:2143-56. doi:10.1093/molbev/msz138.

34. Clark AG, Eisen MB, Smith DR, Bergman CM, Oliver B, Markow TA, et al. Evolution of genes and genomes on the Drosophila phylogeny. Nature. 2007;450 7167:203-18. doi:10.1038/nature06341.

35. Zhan S, Merlin C, Boore Jeffrey L and Reppert Steven M. The Monarch Butterfly Genome Yields Insights into Long-Distance Migration. Cell. 2011;147 5:1171-85. doi:https://doi.org/10.1016/j.cell.2011.09.052.

36. Tribolium Genome Sequencing C, Richards S, Gibbs RA, Weinstock GM, Brown SJ, Denell R, et al. The genome of the model beetle and pest Tribolium castaneum. Nature. 2008;452 7190:949-55. doi:10.1038/nature06784.

37. Wallberg A, Bunikis I, Vinnere Pettersson O, Mosbech M-B, Childers A, Evans J, et al. A hybrid de novo genome assembly of the honeybee, Apis mellifera , with chromosome-length scaffolds. BMC Genomics 20, 275 (2019). https://doi.org/10.1186/s12864-019-5642-0

38. Emms DM and Kelly S. OrthoFinder: solving fundamental biases in whole genome comparisons dramatically improves orthogroup inference accuracy. Genome Biol. 2015;16:157. doi:10.1186/s13059-015-0721-2.

39. Edgar R. MUSCLE: Multiple Sequence Alignment with High Accuracy and High Throughput. Nucleic acids research. 2004;32:1792-7. doi:10.1093/nar/gkh340.

40. Stamatakis A. RAxML version 8: a tool for phylogenetic analysis and post-analysis of large phylogenies. Bioinformatics. 2014;30 9:1312-3. doi:10.1093/bioinformatics/btu033.

41. Yang Z. PAML 4: phylogenetic analysis by maximum likelihood. Mol Biol Evol. 2007;24

8:1586-91. doi:10.1093/molbev/msm088.

42. He Z, Zhang H, Gao S, Lercher MJ, Chen W-H and Hu S. Evolview v2: an online visualization and management tool for customized and annotated phylogenetic trees. Nucleic Acids Research. 2016;44 W1:W236-W41. doi:10.1093/nar/gkw370 %J Nucleic Acids Research.

43. Chen S, Zhou Y, Chen Y and Gu J. fastp: an ultra-fast all-in-one FASTQ preprocessor. Bioinformatics. 2018;34 17:i884-i90. doi:10.1093/bioinformatics/bty560 %J Bioinformatics.

44. Terhorst J, Kamm JA and Song YS. Robust and scalable inference of population history from hundreds of unphased whole genomes. Nat Genet. 2017;49 2:303-9. doi:10.1038/ng.3748.

45. Bachtrog D, Kirkpatrick M, Mank JE, McDaniel SF, Pires JC, Rice W, et al. Are all sex chromosomes created equal? Trends in Genetics. 2011;27 9:350-7. doi:https://doi.org/10.1016/j.tig.2011.05.005.

46. Bachtrog D, Mank JE, Peichel CL, Kirkpatrick M, Otto SP, Ashman TL, et al. Sex determination: why so many ways of doing it? PLoS Biol. 2014;12 7:e1001899. doi:10.1371/journal.pbio.1001899.

47. Rowe L, Chenoweth SF and Agrawal AF. The Genomics of Sexual Conflict. Am Nat. 2018;192 2:274-86. doi:10.1086/698198.

48. Walia GK, Kaur H and Kaur J. Karyotypic Variations in the Chromosome Complement of Pantala flavescens (Fabricius) of the Family Libellulidae (Anisoptera: Odonata). Cytologia. 2011;76 3:301-7. doi:10.1508/cytologia.76.301.

49. Li Y, Zhang B and Moran NA. The Aphid X Chromosome Is a Dangerous Place for Functionally Important Genes: Diverse Evolution of Hemipteran Genomes Based on Chromosome-Level Assemblies. Mol Biol Evol. 2020;37 8:2357-68. doi:10.1093/molbev/msaa095.

50. Castillo ER, Marti DA and Bidau CJ. Sex and Neo-Sex Chromosomes in Orthoptera: A Review*. Journal of Orthoptera Research. 2010;19 2:213-31. doi:10.1665/034.019.0207.

51. Li Y, Park H, Smith TE, Moran NA and Singh N. Gene Family Evolution in the Pea Aphid Based on Chromosome-Level Genome Assembly. Molecular Biology and Evolution. 2019;36 10:2143-56. doi:10.1093/molbev/msz138.

52. Bolton PE, Rollins LA, Brazill-Boast J, Maute KL, Legge S, Austin JJ, et al. Genetic diversity through time and space: diversity and demographic history from natural history specimens and serially sampled contemporary populations of the threatened Gouldian finch (Erythrura gouldiae). Conservation Genetics. 2018;19 3:737-54. doi:10.1007/s10592-018-1051-1.

53. Wang J, Santiago E and Caballero A. Prediction and estimation of effective population size. Heredity. 2016;117 4:193-206. doi:10.1038/hdy.2016.43.

54. Hewitt G. The genetic legacy of the Quaternary ice ages. Nature. 2000;405 6789:907-13. doi:10.1038/35016000.

55. Clark PU, Dyke AS, Shakun JD, Carlson AE, Clark J, Wohlfarth B, et al. The Last Glacial Maximum. Science. 2009;325 5941:710-4. doi:10.1126/science.1172873.

56. Zhao S, Zheng P, Dong S, Zhan X, Wu Q, Guo X, et al. Whole-genome sequencing of

giant pandas provides insights into demographic history and local adaptation. Nat Genet. 2013;45 1:67-71. doi:10.1038/ng.2494.

57. Liu Q, Ruan J, Shi D, Chen B, Qian Q, Ye G, et al. Understanding divergent domestication traits from the whole-genome sequencing of swamp- and river-buffalo populations. National Science Review. 2020;7 3:686-701. doi:10.1093/nsr/nwaa024.

58. Feng S, Fang Q, Barnett R, Li C, Han S, Kuhlwilm M, et al. The Genomic Footprints of the Fall and Recovery of the Crested Ibis. Curr Biol. 2019;29 2:340-9 e7. doi:10.1016/j.cub.2018.12.008.

59. Liu HW, Jiang F, Wang S, Wang HC,Wang A, Zhao H, Xu D, Yang B, Fan W. Supporting data for "Chromosome-level genome of the globe skimmer dragonfly (Pantala flavescens)". GigaScience Database. http://dx.doi.org/10.5524/100972 .

Figure2
Click here to access/download;Figure;Figure2.pdf

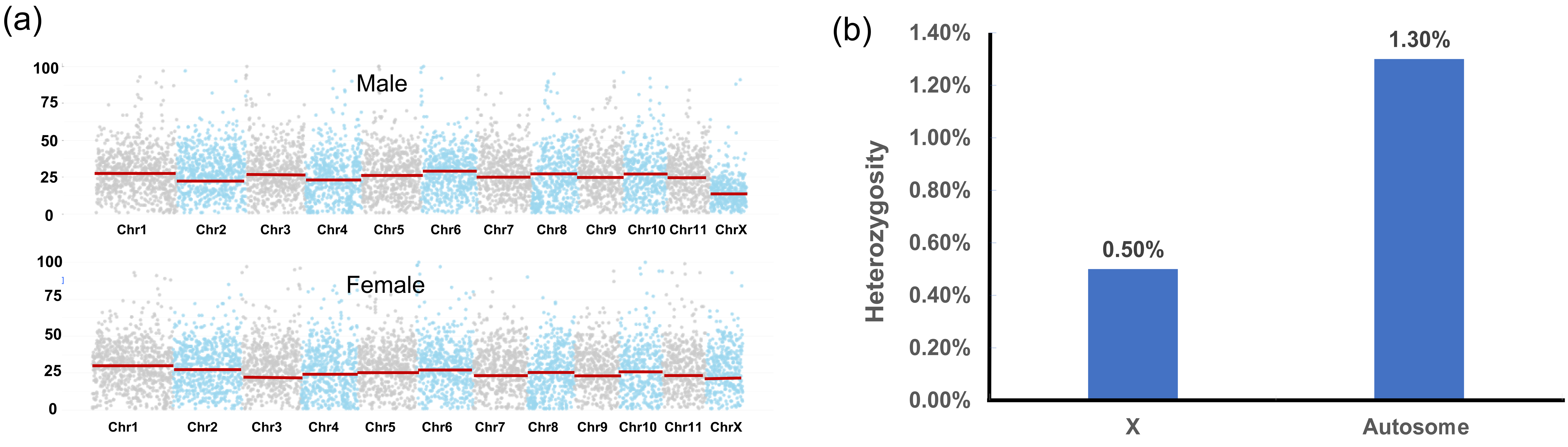

Click here to access/download;Figure;Figure3.pdf

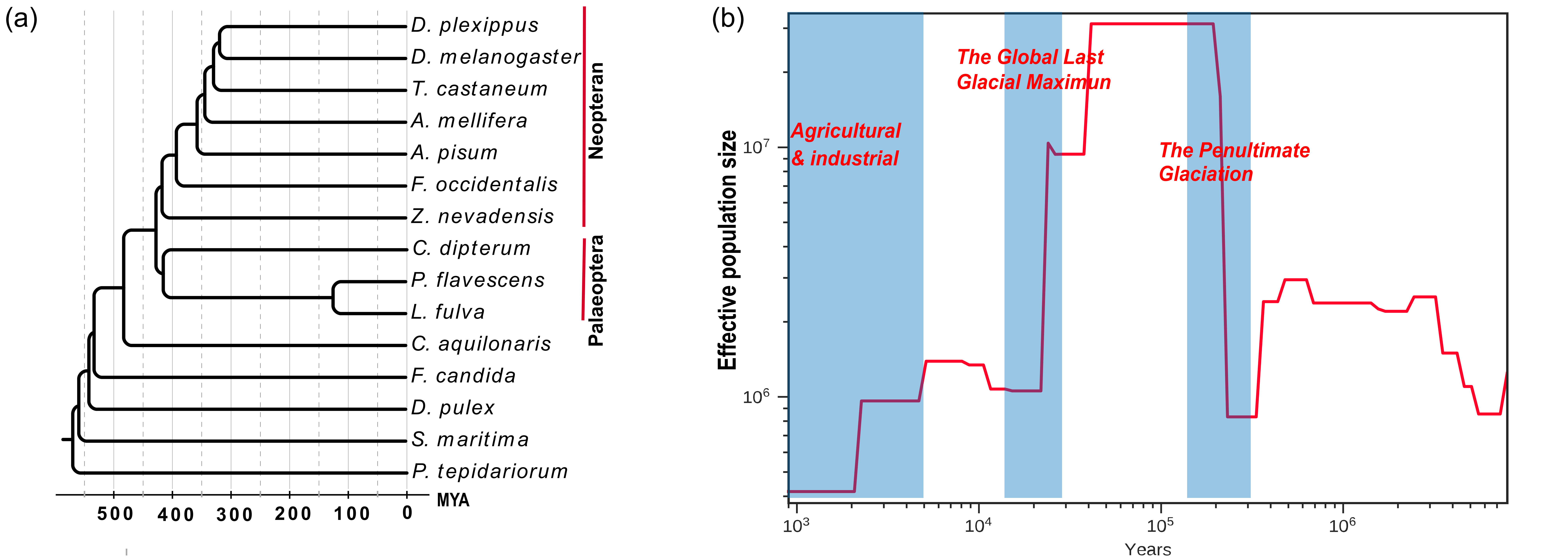

Click here to access/download;Figure;Figure1-revised.pdf

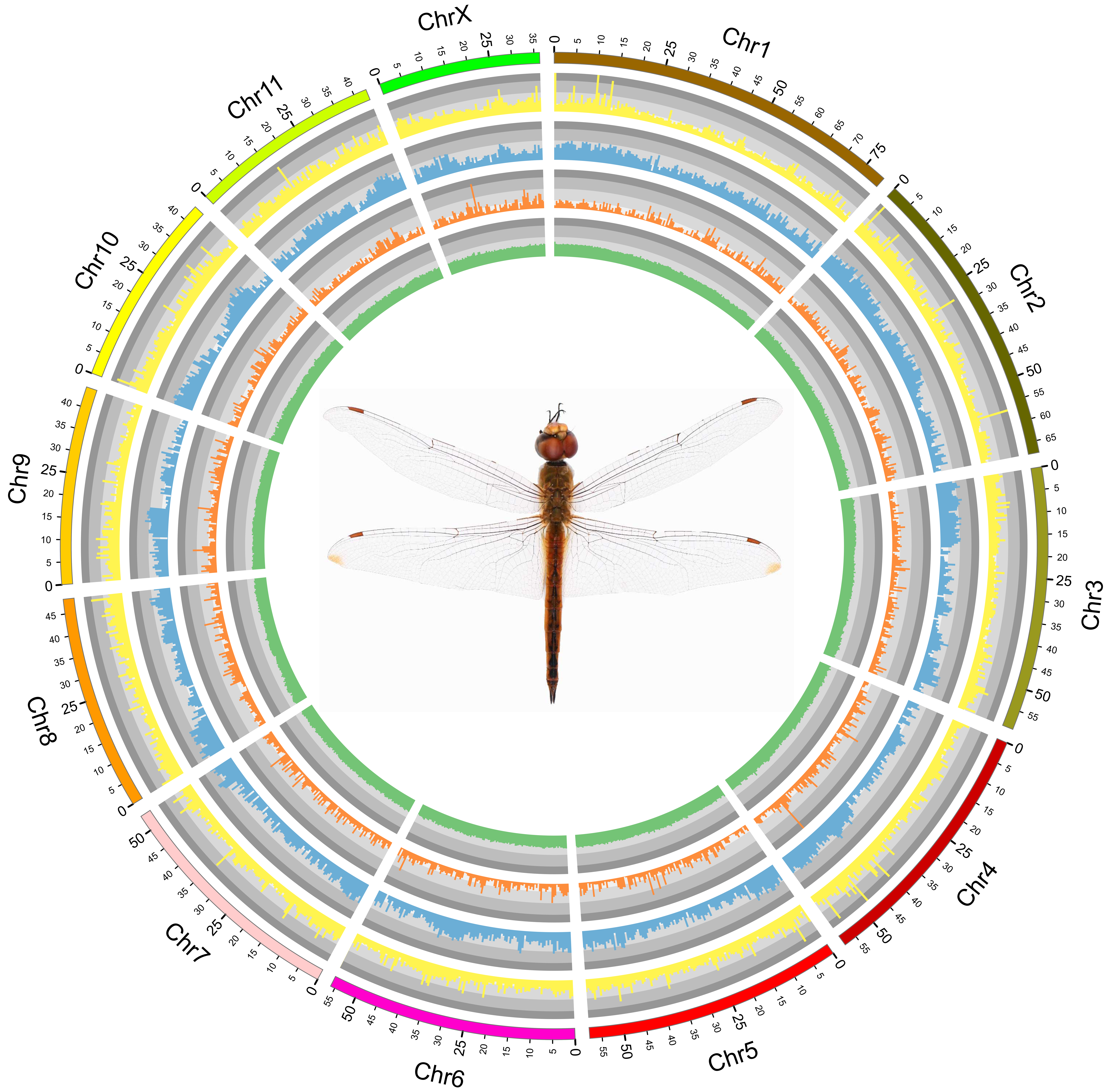

Click here to access/download
Supplementary Material
Supplementary Information-revised.docx

Click here to access/download;Personal Cover;Cover letter-revised.docx

Dear Editor,

We are very grateful to your efforts to review our manuscript entitled "Chromosome - level genome of the globe skimmer dragonfly *(Pantala flavescens)*". We have checked our manuscript carefully, and made improvements on both analyses and presentations according to your and the reviewers' suggestions. Especially, we generated a new gene set, improving BUSCO complete ratio from 98.1% to 98.9%.

I believe that the revised manuscript is more informative, clear and compelling. The revised manuscript and the "responses to reviewers" have been uploaded online. If I can provide you with any further information or assistance, please feel free to contact me at fanwei@caas.cn.

Respectfully,

Fan wei

Guangdong Laboratory for Lingnan Modern Agriculture (Shenzhen Branch), Genome Analysis Laboratory of the Ministry of Agriculture and Rural Affairs, Agricultural Genomics Institute at Shenzhen, Chinese Academy of Agricultural Sciences, Shenzhen, Guangdong, 518120, China

Email: fanwei@caas.cn
