# Supplementary material for: Chromosome-level genome of the globe skimmer dragonfly (Pantala flavescens)
Source: Gigascience. 2022 Apr 4;11:giac009. doi: 10.1093/gigascience/giac009 (PMC8978299; doi:10.1093/gigascience/giac009)
Supplement: giac009_Supplemental_File [file giac009_supplemental_file.docx]

**Table S1: Sequencing statistics**

|  | Gender | Sequencing platform | Data volume |
| --- | --- | --- | --- |
| Genome sequence of HiFi | Female | CCS | two cells (50 Gbp) |
| Genome sequence of Illumina | Female and Male | Illumina | 40 Gbp |
| Iso-Seq | Female and Male | Pacbio | 32 Gbp |
| Hi-C | Male | Illumina NovaSeq 6000 | 170 Gbp |

**Table S2: Statistics of gene predictions from different gene prediction methods**

|  | AUGUSTUS | Homology | Trans  (Illumina) | Trans  (Pacbio) | EVM |
| --- | --- | --- | --- | --- | --- |
| Total gene number | 15,385 | 6,165 | 9,678 | 6,051 | 15,354 |
| Average CDS length | 1,644 | 1,536 | 1,475 | 1,485 | 1,528 |
| Average exon number | 7.9 | 7.8 | 7.3 | 6.8 | 7.1 |
| BUSCO assessment | 97.4% | 58.6% | 75.4% | 64.4% | 98.9% |

**Table S3: Function annotation of gene set.**

| **Database** | **Number of genes** | **Ratio** |
| --- | --- | --- |
| NR | 12,995 | 85% |
| Uniprot | 12,417 | 81% |
| KEGG | 10,346 | 67% |
| Interproscan | 13,240 | 86% |
| All | 14,024 | 91% |


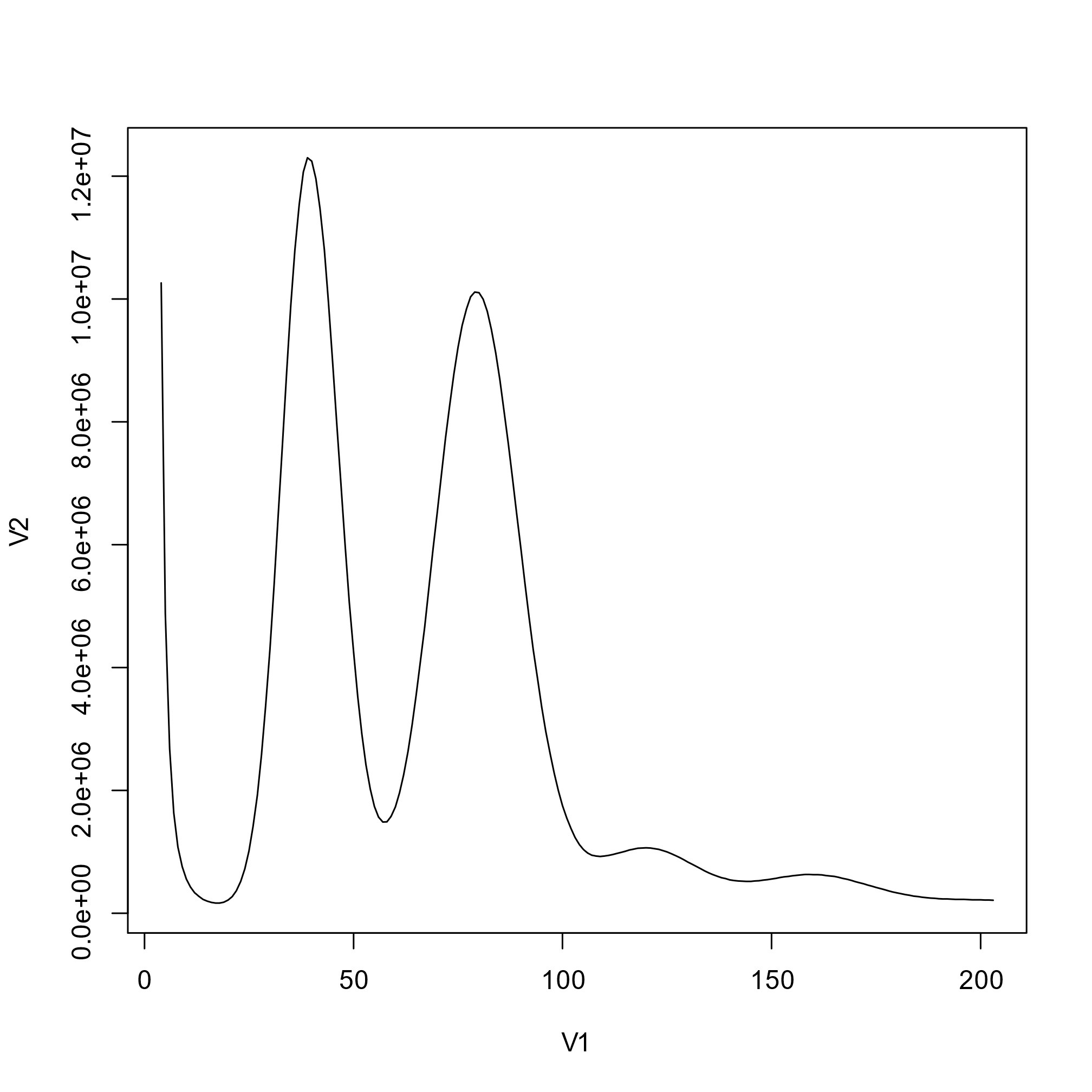


Main peak(depth=80)

**Figure S1: Estimated genome size using 17-kmer.** **We used error corrected Illumina reads from the short insert-size libraries to calculate the k-mer frequency. The peak depth of this curve was 80. The estimated genome size of *Pantala flavescens* was 663Mbp.**


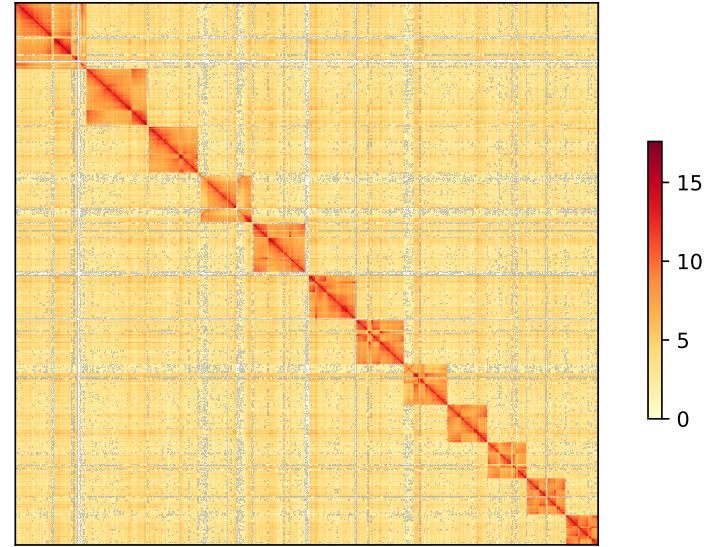


**Figure S2: HiC heatmap of LACHESIS.** **A genome-wide contact matrix from Hi-C data between each pair of the 12 chromosomes using a 1 Mb window size.**

**Figure S3: Major subfamilies of transposable element in *Pantala flavescens* genome..SINEs:** **Short interspersed nuclear elements. LINEs:** **Longinterspersed nuclear element. LTR:** **Long terminal repeats.DNA:DNA transposon.**
