# Supplementary material for: Chromosome-level genome of the globe skimmer dragonfly (Pantala flavescens)
Source: Gigascience. 2022 Apr 4;11:giac009. doi: 10.1093/gigascience/giac009 (PMC8978299; doi:10.1093/gigascience/giac009)
Supplement: giac009_Response_to_Reviewer_Comments_Revision_1 [file giac009_response_to_reviewer_comments_revision_1.pdf]

Author's Response To Reviewer Comments

Close

GIGA-D-21-00299
Chromosome-level genome of the globe skimmer dragonfly (Pantala flavescens)
hangwei liu; fan jiang; sen wang; hengchao wang; anqi wang; hanbo zhao; dong xu; boyuan yang; wei fan
GigaScience

Dear Dr. Fan,

Your manuscript "Chromosome-level genome of the globe skimmer dragonfly (Pantala flavescens)" (GIGA-D-21-00299) has been assessed by our reviewers. Based on these reports, and my own assessment as Editor, I am pleased to inform you that it is potentially acceptable for publication in GigaScience, once you have carried out some essential revisions suggested by our reviewers.

Their reports, together with any other comments, are below. Please also take a moment to check our website at https://www.editorialmanager.com/giga/ for any additional comments that were saved as attachments.

In addition, please register any new software application in the bio.tools and SciCrunch.org databases to receive RRID (Research Resource Identification Initiative ID) and biotoolsID identifiers, and include these in your manuscript. This will facilitate tracking, reproducibility and re-use of your tool.

Once you have made the necessary corrections, please submit a revised manuscript online at:

https://www.editorialmanager.com/giga/

If you have forgotten your username or password please use the "Send Login Details" link to get your login information. For security reasons, your password will be reset.

Please include a point-by-point within the 'Response to Reviewers' box in the submission system. Please ensure you describe additional experiments that were carried out and include a detailed rebuttal of any criticisms or requested revisions that you disagreed with. Please also ensure that your revised manuscript conforms to the journal style, which can be found in the Instructions for Authors on the journal homepage. If the data and code has been modified in the revision process please be sure to update the public versions of this too.

The due date for submitting the revised version of your article is 01 Feb 2022.

We look forward to receiving your revised manuscript soon.

Best wishes,

Hongfang Zhang
GigaScience
www.gigasciencejournal.com

Reviewer reports:
Reviewer #1: This paper describes the sequencing, assembly and annotation of a dragonfly (P. flavescens) genome. Very importantly, the assembly is at chromosome level, making it extremely useful for anyone who wants to run reliable comparative genomics analyses. Effectively, the authors have produced a reference palaeopteran genome. In addition to generating this very useful genomic resource, the authors have run a couple of analyses such as the study of the effective population size along evolutionary time, and a phylogenomic analysis.

I did note that the BUSCO score of the gene set is less than that of the genome assembly. The truth is that the difference is small; only 0.7%. Nevertheless, this means that the gene prediction pipeline doesn't work very well. If BUSCO was able to find 98.8% of conserved genes, then a usually more "sophisticated" gene prediction pipeline should be able to find at least the same number of genes (if not more). Quite often these genes could be missed because they're marked as repeats by RepeatModeler. For this reason, manual inspection of the "unknown" repeats is encouraged in order to exclude "normal" genes that look like repeats (e.g. duplicated genes). For the purposes of this Data Note and since the difference is small, I believe that it might not be necessary for the authors to improve the predicted gene set. If they decide to do so, then that would be great for anyone who would use their data in the future.
Response: Thanks for the reviewer's kind suggestion, and we have refined the repeat annotation and re-run the gene annotation.

To avoid protein-coding genes being marked as repeats, we aligned the 260 repeat sequences of 'unknown' type to NR database by blastx (v2.7.1+) using 1e−5 as cutoff, and 53 of them were found to have homology with known non-TE protein-coding genes, which were filtered out of RepeatModeler de novo library. Repeatmasker was then used to find TEs based on the filtered de novo TE library. After that, we re-run the gene annotation pipeline, and 15,354 genes were annotated. This time, we used the latest BUSCO (v5.2.2) version with insecta_odb10 to assess the gene set, and showed that 98.9% genes was complete, higher than the value of genome 96.9%.

The method of genome annotation in line138 has also been updated.

Regarding the methodology and results on the effective population size, I'm not an expert and therefore I cannot judge their correctness. As for the phylogenomic analysis, it was properly done and the results obtained are correct.

Last, while I was able to have a look at the deposited raw reads (PacBio and Illumina) in SRA, I wasn't able to see the deposited assembly and gene set. I know that it is possible to generate "reviewer links" for SRA submissions, but I'm not sure if it is also the case for submitted genome assemblies and gene sets. If it is possible, could the authors generate such a link?
Response: Now the assembly has been released in NCBI (https://www.ncbi.nlm.nih.gov/genome/103548), and the gene set has also been uploaded to NCBI. These data are also available in AGIS ftp: ftp://ftp.agis.org.cn/~fanwei/Pantala_flavscens/

Some minor corrections:

- Methods -> Evolutionary analysis: In the beginning of the section, where you mention the species you used, Zootermopsis is mentioned twice.
Response: We have revised it.

- Table S1: in the last column of the table, all numbers are "Gbp", not "G". The same is true for the numbers mentioned in the last column of Table 1; they should be "Mbp" and "Kbp".
Response: We have revised it.
- Table S2: I presume that the second column refers to homology-based prediction. If true, then please change the title of the column to "Homology", because "Homo" is confusing.
Response: We have revised "Homo" to "Homology".
- Table S3: "Counts" doesn't adequately describe the numbers in this column. Maybe something like "Number of genes with significant similarity" would be more appropriate.
Response: We have revised "Counts" to "Number of genes".

Reviewer #2: In this manuscript Hangwei Liu and co-authors report the sequencing and genome assembly of the dragonfly species, Pantala flavescens.
This 662 Mb genome assembly is distributed in 12 chromosomes. Genome annotation resulted in almost 15,000 genes, corresponding to a high degree of completeness using BUSCO databases. The authors also identify the sexual chromosome (X) by comparing the ratio of sequenced reads between male and females. Finally, they performed an estimation of the demographic history and detected three events of population decline.
The work will be relevant for the fields of evolutionary biology, evolutionary genomics and researchers working in the evolution of insects. Although I am missing further analyses of different genome features that would increase the scope of the manuscript- especially those mentioned in the introduction, such as the appearance of wings in insects or the study of gene families important for migration, insect ecology,

etc. -, a chromosome grade genome of an Odonata species is of great value for the community. Therefore, I recommend the publication of this manuscript as a Data Note in GigaScience. However, I have some comments that should be addressed prior publication:

1. Since this is a Data note manuscript, a more detailed methodology would be recommended. It is not clear to me how many males or females have been used for the different sequencing protocols: for the PacBio Hifi one female was used, what about the Illumina and the RNA-seq?
Response: We have revised the sequencing protocols in method to make it clearer.

line 96: "For Illumina sequencing, a short paired-end DNA library with a 400 bp insert size from female and male adult P. flavescens" have been revised to "For Illumina sequencing, a short paired-end DNA library with a 400 bp insert size from a female adult and a male adult P. flavescens".

line 105: We revised "Total RNA was extracted separately from females and males and then mixed" to "Total RNA was extracted from a female adult and a male adult and then mixed to generate the libraries."

Line 124: We revised "A total of 170 Gb of Hi-C paired-end reads were generated" to "A total of 170 Gb of Hi-C paired-end reads were generated from a female adult"

- "Insects were removed from the intestine to avoid bacterial contamination,":
Is it just bacterial contamination or also contamination from prey (insects?) genomes? Please clarify.
Response: The contamination includes bacterial and prey genomes. So, we have revised line93 "Insects were removed from the intestine to avoid bacterial contamination" to "Insects were removed from the intestine to avoid contamination from bacterial and prey genomes".

- "Total RNA was extracted separately from females and males and then mixed": Does it mean that the total RNA was mixed to generate the libraries or that libraries and sequencing was done independently for male and females and the data was merged for the subsequent analyses?
Response: Our meaning is that total RNA was mixed to generate the libraries. And to make it clearer, we revised line 105"Total RNA was extracted separately from females and males and then mixed" to "Total RNA was extracted from a female adult and a male adult and then mixed to generate the libraries."

2. BUSCO analysis and comparisons. Perhaps a table with the percentage of completeness for the different arthropods would be clearer to visualize instead of the plot with the horizontal bars.
Response: We have changed Figure1b to a Table2.

- Also regarding the BUSCO data, it would be good if you listed the source of these numbers from other arthropods, referencing the primary articles, especially in the case of Ladona fulva (https://doi.org/10.15482/USDA.ADC/1503790.) and Cloeon dipterum (https://doi.org/10.1038/s41467-020-16284-8), since they are both respectively used as the most closely related genome or as an outgroup within the Paleoptera lineage along the entire manuscript. Actually, for the C. dipterum data, the original paper reported 96.9% complete and 1.3% fragmented whereas according to the figure 1b, C. dipterum genome has more than 97% of complete and around 1% of fragmented sets, could you explain this minor discrepancy?
Response: Thanks for the suggestion, and we have revised the reference and cited the primary articles about other genomes used in this manuscript.

The BUSCO assessments were performed by ourselves using BUSCO (5.2.2), not copied from reference papers. So, the discrepancy of C. dipterum BUSCO assessment between the original paper and this manuscript might be caused by different versions of BUSCO and insecta_odb.

3. "Genomic resources for insects available in public databases are mainly focused on dipteran flies, lepidopterans and hymenopterans":
While it is true that historically available genomes belong mainly to Diptera, Lepidoptera and other holometabola, genome projects for hemimetabolous insects have been developed recently, thus acknowledging the existence of these efforts and new genomes would be desirable: see for instance crickets: Ylla et al. 2021(https://doi.org/10.1038/s42003-021-02197-9), Ephemeroptera: Almudi et al. 2020 (https://doi.org/10.1038/s41467-020-16284-8), damselfly: Ioannidis et al. 2017 (https://doi.org/10.1093/gbe/evx006), Sinella curviseta, collembola: Zhang et al. 2019 (https://doi.org/10.1093/gbe/evz013), giant collembolan: Wu et al. 2017

(https://doi.org/10.1186/s12864-017-4197-1), water strider: Armisén et al. 2018 (https://doi.org/10.1186/s12864-018-5163-2), cockroach: Harrison et al. 2018 (https://doi.org/10.1038/s41559-017-0459-1), among many others.
Response: Thanks for the suggestion, we have revised
line67 “Genomic resources for insects available in public databases are mainly focused on dipteran flies, lepidopterans and hymenopterans, most of which are sanitary or agricultural pests. They do not capture the profile of whole insects, hindering the study of insect evolution.”
into
“Genomic resources for insects available in public databases include dipteran flies, lepidopterans, hymenopterans, blattarias, and so on. However, only four genomes of Odonata species with low continuity have been released, and a high-quality genome of Odonata species is necessary for research.”.

4. The text needs some proofreading, I detected some typos or sentences that sound a bit odd:

- 4a. Page 4:
"… is the most parasitoid species used worldwide [3]."
I think that the authors probably meant:
"…is the parasitoid species most used worldwide"
Response: The sentence has been revised according to the reviewer’s kind suggestion.

- 4b. Page 5:
"of Palaeopteran insects, which is the first winged insect and the sister of Neopterans"
change to:
"of Palaeopteran insects, which are the first winged insects and the sister group of Neopterans "
Response: The sentence has been revised according to the reviewer’s kind suggestion.

- 4c. Page 5:
"Powerful flight capabilities with varied wing dimorphism facilitate migration, escape and mating of winged insect (Pterygota), as well as more resources and habitats can be occupied by Pterygota insects." instead of "dimorphism facilitate winged insect (Pterygota) migration, escape and mating, and more resources and habitats can be occupied by Pterygota insects."
Response: The sentence has been revised according to the reviewer’s kind suggestion.

- 4d. Page 5:
"Despite the attractiveness of this group for evolutionary genomic analysis, efforts have lagged behind for other insect orders." instead of "Despite the attractiveness of this group for evolutionary genomic analysis, efforts have lagged behind those of other insect orders."
Response: The sentence has been revised according to the reviewer’s kind suggestion.

- 4e. Page 8:
"Zootermopsis nevadensis, Zootermopsis nevadensis," appears duplicated.
Response: The sentence has been revised according to the reviewer’s kind suggestion.

- 4f. Page 9:
"Therefore, the genome assembly of P. flavescens is a high-quality and highly contiguous genome." instead of "Therefore, the genome assembly of P. flavescens presents is highly contiguous and has a high sequence quality."
Response: The sentence has been revised according to the reviewer’s kind suggestion.

5. Supplementary figure legends are missing, a brief description of the figure should be added besides the title of the figure.
Response: We have added figure legends and a brief description to Supplementary figures.

Please also take a moment to check our website at for any additional comments that were saved as attachments. Please note that as GigaScience has a policy of open peer review, you will be able to see the names of the reviewers.

Close
