# Supplementary material for: Chromosome-level genome of the globe skimmer dragonfly (Pantala flavescens)
Source: Gigascience. 2022 Apr 4;11:giac009. doi: 10.1093/gigascience/giac009 (PMC8978299; doi:10.1093/gigascience/giac009)
Supplement: giac009_Reviewer_1_Report_Original_Submission — Panagiotis Ioannidis -- 10/31/2021 Reviewed [file giac009_reviewer_1_report_original_submission.pdf]

Reviewer Report

Title: Chromosomeâ€level genome of the globe skimmer dragonfly (Pantala flavescens)

Version: Original Submission **Date:** 10/31/2021

Reviewer name: Panagiotis Ioannidis

Reviewer Comments to Author:

This paper describes the sequencing, assembly and annotation of a dragonfly (P. flavescens) genome. Very importantly, the assembly is at chromosome level, making it extremely useful for anyone who wants to run reliable comparative genomics analyses. Effectively, the authors have produced a reference palaeopteran genome. In addition to generating this very useful genomic resource, the authors have run a couple of analyses such as the study of the effective population size along evolutionary time, and a phylogenomic analysis.

I did note that the BUSCO score of the gene set is less than that of the genome assembly. The truth is that the difference is small; only 0.7%. Nevertheless, this means that the gene prediction pipeline doesn't work very well. If BUSCO was able to find 98.8% of conserved genes, then a usually more "sophisticated" gene prediction pipeline should be able to find at least the same number of genes (if not more). Quite often these genes could be missed because they're marked as repeats by RepeatModeler. For this reason, manual inspection of the "unknown" repeats is encouraged in order to exclude "normal" genes that look like repeats (e.g. duplicated genes). For the purposes of this Data Note and since the difference is small, I believe that it might not be necessary for the authors to improve the predicted gene set. If they decide to do so, then that would be great for anyone who would use their data in the future.

Regarding the methodology and results on the effective population size, I'm not an expert and therefore I cannot judge their correctness. As for the phylogenomic analysis, it was properly done and the results obtained are correct.

Last, while I was able to have a look at the deposited raw reads (PacBio and Illumina) in SRA, I wasn't able to see the deposited assembly and gene set. I know that it is possible to generate "reviewer links" for SRA submissions, but I'm not sure if it is also the case for submitted genome assemblies and gene sets. If it is possible, could the authors generate such a link?

Some minor corrections:

- Methods -> Evolutionary analysis: In the beginning of the section, where you mention the species you used, Zootermopsis is mentioned twice.
- Table S1: in the last column of the table, all numbers are "Gbp", not "G". The same is true for the numbers mentioned in the last column of Table 1; they should be "Mbp" and "Kbp".
- Table S2: I presume that the second column refers to homology-based prediction. If true, then please change the title of the column to "Homology", because "Homo" is confusing.
- Table S3: "Counts" doesn't adequately describe the numbers in this column. Maybe something like "Number of genes with significant similarity" would be more appropriate.

Level of Interest

Please indicate how interesting you found the manuscript: Choose an item.

Quality of Written English

Please indicate the quality of language in the manuscript: Choose an item.

Declaration of Competing Interests

Please complete a declaration of competing interests, considering the following questions:

- Have you in the past five years received reimbursements, fees, funding, or salary from an organisation that may in any way gain or lose financially from the publication of this manuscript, either now or in the future?
- Do you hold any stocks or shares in an organisation that may in any way gain or lose financially from the publication of this manuscript, either now or in the future?
- Do you hold or are you currently applying for any patents relating to the content of the manuscript?
- Have you received reimbursements, fees, funding, or salary from an organization that holds or has applied for patents relating to the content of the manuscript?
- Do you have any other financial competing interests?
- Do you have any non-financial competing interests in relation to this paper?

If you can answer no to all of the above, write 'I declare that I have no competing interests' below. If your reply is yes to any, please give details below.

I declare that I have no competing interests

I agree to the open peer review policy of the journal. I understand that my name will be included on my report to the authors and, if the manuscript is accepted for publication, my named report including any attachments I upload will be posted on the website along with the authors' responses. I agree for my report to be made available under an Open Access Creative Commons CC-BY license (http://creativecommons.org/licenses/by/4.0/). I understand that any comments which I do not wish to be included in my named report can be included as confidential comments to the editors, which will not be published.

Choose an item.

To further support our reviewers, we have joined with Publons, where you can gain additional credit to further highlight your hard work (see: https://publons.com/journal/530/gigascience). On publication of this paper, your review will be automatically added to Publons, you can then choose whether or not to claim your Publons credit. I understand this statement.

Yes Choose an item.
