# Supplementary material for: Chromosome-level genome of the globe skimmer dragonfly (Pantala flavescens)
Source: Gigascience. 2022 Apr 4;11:giac009. doi: 10.1093/gigascience/giac009 (PMC8978299; doi:10.1093/gigascience/giac009)
Supplement: giac009_Reviewer_2_Report_Original_Submission — Isabel Almudi -- 11/3/2021 Reviewed [file giac009_reviewer_2_report_original_submission.pdf]

Reviewer Report

Title: Chromosomeâ€level genome of the globe skimmer dragonfly (Pantala flavescens)

Version: Original Submission **Date:** 11/3/2021

Reviewer name: Isabel Almudi

Reviewer Comments to Author:

In this manuscript Hangwei Liu and co-authors report the sequencing and genome assembly of the dragonfly species, Pantala flavescens.

This 662 Mb genome assembly is distributed in 12 chromosomes. Genome annotation resulted in almost 15,000 genes, corresponding to a high degree of completeness using BUSCO databases. The authors also identify the sexual chromosome (X) by comparing the ratio of sequenced reads between male and females. Finally, they performed an estimation of the demographic history and detected three events of population decline.

The work will be relevant for the fields of evolutionary biology, evolutionary genomics and researchers working in the evolution of insects. Although I am missing further analyses of different genome features that would increase the scope of the manuscript- especially those mentioned in the introduction, such as the appearance of wings in insects or the study of gene families important for migration, insect ecology, etc. -, a chromosome grade genome of an Odonata species is of great value for the community. Therefore, I recommend the publication of this manuscript as a Data Note in GigaScience. However, I have some comments that should be addressed prior publication:

1. Since this is a Data note manuscript, a more detailed methodology would be recommended. It is not clear to me how many males or females have been used for the different sequencing protocols: for the PacBio Hifi one female was used, what about the Illumina and the RNA-seq?

- "Insects were removed from the intestine to avoid bacterial contamination,":

Is it just bacterial contamination or also contamination from prey (insects?) genomes? Please clarify.

- "Total RNA was extracted separately from females and males and then mixed": Does it mean that the total RNA was mixed to generate the libraries or that libraries and sequencing was done independently for male and females and the data was merged for the subsequent analyses?

2. BUSCO analysis and comparisons. Perhaps a table with the percentage of completeness for the different arthropods would be clearer to visualize instead of the plot with the horizontal bars.

- Also regarding the BUSCO data, it would be good if you listed the source of these numbers from other arthropods, referencing the primary articles, especially in the case of Ladona fulva (https://doi.org/10.15482/USDA.ADC/1503790.) and Cloeon dipterum (https://doi.org/10.1038/s41467-020-16284-8), since they are both respectively used as the most closely related genome or as an outgroup within the Paleoptera lineage along the entire manuscript. Actually, for the C. dipterum data, the original paper reported 96.9% complete and 1.3% fragmented whereas according to the figure 1b, C. dipterum genome has more than 97% of complete and around 1% of fragmented sets, could you explain this minor discrepancy?

3. "Genomic resources for insects available in public databases are mainly focused on dipteran flies,

lepidopterans and hymenopterans":
While it is true that historically available genomes belong mainly to Diptera, Lepidoptera and other holometabola, genome projects for hemimetabolous insects have been developed recently, thus acknowledging the existence of these efforts and new genomes would be desirable: see for instance crickets: Ylla et al. 2021(https://doi.org/10.1038/s42003-021-02197-9), Ephemeroptera: Almudi et al. 2020 (https://doi.org/10.1038/s41467-020-16284-8), damselfly: Ioannidis et al. 2017 (https://doi.org/10.1093/gbe/evx006), Sinella curviseta, collembola: Zhang et al. 2019 (https://doi.org/10.1093/gbe/evz013), giant collembolan: Wu et al. 2017 (https://doi.org/10.1186/s12864-017-4197-1), water strider: ArmisÃ©n et al. 2018 (https://doi.org/10.1186/s12864-018-5163-2), cockroach: Harrison et al. 2018 (https://doi.org/10.1038/s41559-017-0459-1), among many others.
4. The text needs some proofreading, I detected some typos or sentences that sound a bit odd:
- 4a. Page 4:
"… is the most parasitoid species used worldwide [3]."
I think that the authors probably meant:
"…is the parasitoid species most used worldwide"
- 4b. Page 5:
"of Palaeopteran insects, which is the first winged insect and the sister of Neopterans"
change to:
"of Palaeopteran insects, which are the first winged insects and the sister group of Neopterans "
- 4c. Page 5:
"Powerful flight capabilities with varied wing dimorphism facilitate migration, escape and mating of winged insect (Pterygota), as well as more resources and habitats can be occupied by Pterygota insects." instead of "dimorphism facilitate winged insect (Pterygota) migration, escape and mating, and more resources and habitats can be occupied by Pterygota insects."
- 4d. Page 5:
"Despite the attractiveness of this group for evolutionary genomic analysis, efforts have lagged behind for other insect orders." instead of "Despite the attractiveness of this group for evolutionary genomic analysis, efforts have lagged behind those of other insect orders."
- 4e. Page 8:
"Zootermopsis nevadensis, Zootermopsis nevadensis," appears duplicated.
- 4f. Page 9:
"Therefore, the genome assembly of P. flavescens is a high-quality and highly contiguous genome." instead of "Therefore, the genome assembly of P. flavescens presents is highly contiguous and has a high sequence quality."
5. Supplementary figure legends are missing, a brief description of the figure should be added besides the title of the figure.

Level of Interest

Please indicate how interesting you found the manuscript: Choose an item.

Quality of Written English

Please indicate the quality of language in the manuscript: Choose an item.

Declaration of Competing Interests

Please complete a declaration of competing interests, considering the following questions:

- Have you in the past five years received reimbursements, fees, funding, or salary from an organisation that may in any way gain or lose financially from the publication of this manuscript, either now or in the future?
- Do you hold any stocks or shares in an organisation that may in any way gain or lose financially from the publication of this manuscript, either now or in the future?
- Do you hold or are you currently applying for any patents relating to the content of the manuscript?
- Have you received reimbursements, fees, funding, or salary from an organization that holds or has applied for patents relating to the content of the manuscript?
- Do you have any other financial competing interests?
- Do you have any non-financial competing interests in relation to this paper?

If you can answer no to all of the above, write 'I declare that I have no competing interests' below. If your reply is yes to any, please give details below.

I declare that I have no competing interests

I agree to the open peer review policy of the journal. I understand that my name will be included on my report to the authors and, if the manuscript is accepted for publication, my named report including any attachments I upload will be posted on the website along with the authors' responses. I agree for my report to be made available under an Open Access Creative Commons CC-BY license (http://creativecommons.org/licenses/by/4.0/). I understand that any comments which I do not wish to be included in my named report can be included as confidential comments to the editors, which will not be published.

Choose an item.

To further support our reviewers, we have joined with Publons, where you can gain additional credit to further highlight your hard work (see: https://publons.com/journal/530/gigascience). On publication of this paper, your review will be automatically added to Publons, you can then choose whether or not to claim your Publons credit. I understand this statement.

Yes Choose an item.
